# Duloxetine for postoperative pain management in total hip and knee arthroplasty: a systematic review and meta-analysis

**DOI:** 10.1186/s13018-026-07176-6

**Published:** 2026-09-02

**Authors:** Abdelrahman Ibrahim, Mohamed Abdelnabi, Reda Ali Sheta

**Affiliations:** Department of Orthopedic Surgery, Al-Ahrar Teaching Hospital, Al-Montazah, Zagazig, Al-Sharqia Governorate 7141360 Egypt

**Keywords:** Duloxetine, Total knee arthroplasty, Total hip arthroplasty, Postoperative pain, Opioid-sparing, Multimodal analgesia, Meta-analysis

## Abstract

**Background:**

Postoperative pain following total hip arthroplasty (THA) and total knee arthroplasty (TKA) drives opioid dependence. Perioperative duloxetine is an option as a multimodal analgesic adjunct, yet its efficacy and safety in this population are not fully defined.

**Methods:**

This systematic review and meta-analysis compared perioperative duloxetine to placebo in THA and TKA patients, following the Preferred Reporting Items for Systematic Reviews and Meta-Analyses (PRISMA) guidelines and registered with the International Prospective Register of Systematic Reviews (PROSPERO) (CRD420261294277). The search strategy spanned PubMed, Web of Science, and Scopus. The Cochrane Library was also queried from inception to March 2, 2026. The primary outcomes were Visual Analog Scale (VAS) pain scores at rest and during ambulation; secondary outcomes included postoperative opioid consumption and the incidence of adverse effects. VAS and Numeric Rating Scale (NRS) pain scores were converted to a common 0–100 mm scale for pooling and expressed as mean differences (MD) with 95% confidence intervals (CI); opioid consumption was standardized to morphine milligram equivalents (MME) and expressed as MD in MME. Dichotomous data were expressed as risk ratios (RR).

**Results:**

Thirteen randomized controlled trials (RCTs) (n = 1176) were included. Duloxetine significantly reduced pain at rest (MD − 5.09 mm, 95% CI − 8.76 to − 1.43, *P* = 0.006) and during ambulation (MD − 4.55 mm, 95% CI − 8.71 to −0.40, *P* = 0.03) at 24 h. Analgesic benefits persisted at 2 weeks (rest pain: MD − 6.48 mm, 95% CI − 11.14 to − 1.82, *P* = 0.006; ambulatory pain: MD − 8.93 mm, 95% CI − 13.16 to − 4.69, *P* < 0.0001) and ≥3 months (rest pain: MD − 2.79 mm, 95% CI − 4.93 to − 0.64, *P* = 0.01; ambulatory pain: MD − 3.76 mm, 95% CI − 6.62 to − 0.89, *P* = 0.01). After standardization to morphine milligram equivalents (MME), statistically significant opioid-sparing effects were observed at 48 h (MD − 22.74 MME, 95% CI − 44.17 to − 1.31, *P* = 0.04, I^2^ = 100%) and 72 h (MD − 10.58 MME, 95% CI − 18.67 to − 2.49, *P* = 0.01); both estimates were subject to extreme heterogeneity, and the 48-h estimate in particular should be interpreted with substantial caution given its very wide interval. Duloxetine significantly reduced the risk of nausea/vomiting (RR 0.73, 95% CI 0.55–0.97, *P* = 0.03); a borderline reduction in fatigue was also observed (RR 0.87, 95% CI 0.76–0.996, *P* = 0.04). Duloxetine increased the risk of drowsiness/somnolence (RR 1.88, *P* = 0.001).

**Conclusions:**

Perioperative duloxetine produces statistically significant, though sub-threshold, analgesic effects across all assessed timepoints and a selective opioid-sparing effect spanning 48 to 72 h postoperatively when incorporated into multimodal analgesia for THA and TKA. All findings are subject to substantial heterogeneity and should be interpreted with caution. Duloxetine significantly reduced the incidence of nausea/vomiting and increased the risk of drowsiness/somnolence, the latter warranting preoperative patient counseling; a borderline reduction in fatigue was also observed. However, on leave-one-out sensitivity analysis, the long-term (≥ 3-month) reduction in pain at rest, the nausea/vomiting reduction, and the fatigue reduction each lost statistical significance upon exclusion of a single high-risk trial (Inamullah 2023) and should be interpreted with corresponding caution.

**Supplementary Information:**

The online version contains supplementary material available at https://doi.org/10.1186/s13018-026-07176-6.

## Introduction

Total hip arthroplasty (THA) and total knee arthroplasty (TKA) are standard surgical interventions for end-stage osteoarthritis that improve joint mechanics and quality of life [1, 2]. For knee osteoarthritis specifically, non-surgical alternatives such as intra-articular mesenchymal stem cell-based therapies provide modest, primarily symptom-modifying improvements in pain and function without a consistent effect on joint structure, so patients with end-stage disease still ultimately progress to arthroplasty [3]. While surgeons in the United States currently perform over 1 million procedures annually, annual demand is projected to reach 1.26 million TKAs and 572,000 THAs by 2030 [2, 4]. Despite the implementation of standardized perioperative care pathways, more than half of patients continue to experience moderate-to-severe pain following total joint replacement, representing a sustained clinical burden [5].

Effective analgesia drives surgical recovery, as it improves patient satisfaction and prevents complications such as delayed mobility and the development of chronic pain [5]. Although opioids were traditional analgesic mainstays, their risks—dependence, respiratory depression, postoperative ileus—motivate opioid-sparing multimodal analgesia (MMA) [5]. In orthopedics, where more than 80% of patients utilize postoperative narcotics, high-dose opioid analgesic protocols are independently associated with increased complications, higher revision rates, and prolonged length of stay [6]. Suboptimal pain control directly hinders functional rehabilitation and increases the likelihood of long-term narcotic dependence. Developing refined, evidence-based strategies therefore remains essential to optimizing the clinical trajectory and improving outcomes for joint replacement candidates [2].

Duloxetine (Cymbalta) is a selective serotonin-norepinephrine reuptake inhibitor (SNRI) with established clinical applications in the management of depression, anxiety, chronic musculoskeletal pain, fibromyalgia, and diabetic peripheral neuropathy [7]. By inhibiting the reuptake of serotonin and norepinephrine, it elevates central neurotransmitter levels and thereby reinforces the descending inhibitory pain pathways within the central nervous system [7]. The analgesic effect of duloxetine is considered pharmacologically distinct from its antidepressant properties, though the degree of pain relief may still depend on a patient’s underlying pain phenotype, including central sensitization or comorbid depressive symptoms [7].The most commonly studied perioperative dose is 60 mg daily. Recent studies have evaluated duloxetine as a perioperative adjunct in multimodal protocols [8]—an extension of its role in chronic pain—used to optimize acute postoperative recovery and mitigate opioid reliance in patients undergoing total joint replacement [9].

Despite the clinical rationale, the evidence for duloxetine in joint arthroplasty remains incomplete. Prior reviews are constrained by small sample sizes—and notably exclude several trials published between 2023 and 2024. The present meta‑analysis incorporates 13 randomized controlled trials (1176 patients) and focuses specifically on THA and TKA populations. It provides an updated view of perioperative benefit and safety.

The objective of this systematic review and meta-analysis of RCTs was to evaluate the efficacy and safety of perioperative duloxetine compared to placebo in patients undergoing THA or TKA. By synthesizing the most current evidence, this study provides a time-resolved analysis of duloxetine’s analgesic and opioid-sparing effects.

## Materials and methods

This meta-analysis was conducted in accordance with the Preferred Reporting Items for Systematic Reviews and Meta-Analyses (PRISMA) Statement (Additional file 1: PRISMA Checklist). The study protocol was registered with the International Prospective Register of Systematic Reviews (PROSPERO) under the registration number CRD420261294277 (available at: https://www.crd.york.ac.uk/PROSPERO/view/CRD420261294277). A comprehensive search was performed in March 2026 across PubMed, Web of Science, Scopus, and the Cochrane Library databases to identify relevant literature. The search strategy employed keywords and Boolean operators including (Dulox* OR Dulot OR DXT OR Cymbalta OR Yentreve OR Drizalma OR Irenka OR Xeristar OR Symbal) AND (Arthroplast* OR Replacement OR Prosthesis). We developed specific search strategies for each database (Additional file 2: Table S1) with no restrictions on date or language. After removing 145 duplicates, 470 records remained.

### Eligibility criteria

Study selection followed the PICO framework. The population (P) included patients undergoing Total Hip Arthroplasty (THA) or Total Knee Arthroplasty (TKA). The intervention (I) was the perioperative administration of duloxetine, compared (C) against a placebo. For trials randomizing patients to more than two arms, only the arm(s) satisfying this placebo-controlled comparison were extracted; the single such case, Imani et al. [10], was a three-arm, double-blind RCT comparing duloxetine, pregabalin, and placebo, from which only the duloxetine and placebo arms were used. Primary outcomes (O) were Visual Analog Scale (VAS) pain scores at rest and during ambulation. Secondary outcomes included postoperative opioid consumption and the incidence of adverse effects such as fatigue, nausea, dizziness, and insomnia. Only full-text, peer-reviewed randomized controlled trials (RCTs) were included. Exclusion criteria comprised non-RCT designs, observational studies, or case series with fewer than 10 patients. Secondary research, letters, protocols, and abstracts were excluded if data were incomplete. Trials lacking baseline characteristics, primary outcome data, or postoperative follow-up were omitted. Studies were excluded for unclear methodology or duplicate publication. Exclusions also applied where the full text remained unavailable.

### Screening and data extraction

Two researchers independently completed the preliminary screening of titles and abstracts. Eligible studies underwent full-text evaluation against the PICO criteria. Discrepancies were resolved by consensus. Two researchers independently extracted data and cross-checked entries to prevent errors. The extracted data points included the first author, year of publication, study design, sample size, participant characteristics, daily duloxetine dose, follow-up duration, pain scores (rVAS and aVAS), opioid consumption, and adverse events. For the adverse event analysis, drowsiness/somnolence was treated as a single composite endpoint; the six contributing studies used varying terminology—some reporting “somnolence”, others “drowsiness” or “night-time drowsiness”—but each trial contributed a single event count per arm, which were pooled under the composite label "drowsiness/somnolence." The risk of bias was independently assessed by two researchers using the Cochrane Risk of Bias 2 (RoB 2) tool. Evaluation domains included the randomization process, deviations from intended interventions, missing outcome data, measurement of the outcome, and selection of the reported result. Discrepancies were resolved by consensus.

### Statistical analysis

Pooled effect estimates, heterogeneity statistics, and forest plots were generated using RevMan (version 5.4); publication bias analyses were performed separately in R Statistical Software, as detailed below. For primary outcomes, time points of 24 h, 2 weeks, and 3 months were selected for VAS scores—opioid consumption was tracked at 24 h, 48 h, 72 h, and 7 days. To address potential temporal heterogeneity within the ≥3-month interval, a subgroup analysis was performed for both rVAS and aVAS outcomes, stratifying the contributing studies according to their precisely reported follow-up timepoint: studies reporting outcomes at exactly 3 months and those at 6 months. Pain intensity was recorded on a 0–10 Visual Analog Scale (VAS) or Numeric Rating Scale (NRS) in twelve trials and on a 0–100 mm VAS in one trial [11]; each trial’s instrument is listed in Table 1. The nine trials using a 0–10 VAS [7, 10, 12–18] and the three trials using a 0–10 NRS [19–21] were linearly converted to the 0–100 mm scale by multiplying the reported value by 10 prior to pooling; Rienstra [11], already reported on a 0–100 mm scale, required no conversion. Continuous variables were then expressed as mean differences (MD) with 95% confidence intervals (CI) on this common 0–100 mm metric; MD is the appropriate effect measure in this context and permits direct comparison against established minimal clinically important difference (MCID) thresholds.

**Table 1:** Baseline characteristics of included studies

Author	Country	Operation	Study design	Blinding method	Sample size			Original pain scale^b^
					Dulox group	Placebo group	Total	
Ho [19]	Singapore	TKA	RCT	Double	23	24	47	NRS (0–10)
YaDeau [20]	USA	TKA	RCT	Triple	53	53	106	NRS (0–10)
Koh [13]	South Korea	TKA	RCT	Double	40	40	80	VAS (0–10)
Kim [14]	South Korea	TKA	RCT	Double	19	20	39	VAS (0–10)
Li [15]	China	THA	RCT	Double	48	48	96	VAS (0–10)
Rienstra [11]	Netherlands	THA/TKA	RCT	None	57	54	111	VAS (0–100)
Yuan [7]	China	TKA	RCT	Double	50	50	100	VAS (0–10)
YaDeau [21]	USA	TKA	RCT	Triple	80	80	160	NRS (0–10)
Ding [12]	China	THA	RCT	Double	34	33	67	VAS (0–10)
Imani [10]^a^	Iran	TKA	RCT	Double	20	20	40	VAS (0–10)
Inamullah [16]	Pakistan	TKA	RCT	NR	70	70	140	VAS (0–10)
Pinsornsak [17]	Thailand	TKA	RCT	Double	42	42	84	VAS (0–10)
Rajani [18]	India	TKA	RCT	Double	53	53	106	VAS (0–10)

TKA, total knee arthroplasty; THA, total hip arthroplasty; RCT, randomized controlled trial; Double, double-blind; Triple, triple-blind; NR, not reported; Dulox, duloxetine; NRS, numeric rating scale; VAS, visual analog scale

^a^Imani et al. [10] was a three-arm trial comparing duloxetine, pregabalin, and placebo; only the duloxetine (n = 20) and placebo (n = 20) arms met this review’s placebo-controlled PICO criteria and are reported here. The pregabalin arm was not extracted and does not contribute to any pooled analysis in this review

^b^All 0–10 VAS/NRS scores were linearly converted to the 0–100 mm scale (raw score × 10) for pooling; Rienstra [11] reported natively on a 0–100 mm scale and required no conversion. See Statistical Analysis for details

For opioid consumption outcomes, all reported values were converted to morphine milligram equivalents (MME) prior to pooling. Studies reporting intravenous morphine [15, 17, 19] were converted using an equianalgesic conversion factor of 3.0 for IV/IM morphine, in accordance with the American Pain Society’s Principles of Analgesic Use in the Treatment of Acute Pain and Cancer Pain [22]; studies that already reported data in oral morphine equivalents [20, 21] or MME directly [7] required no conversion. Opioid consumption values were further classified by reporting basis: per-interval (daily) values, representing consumption during a discrete window (e.g., hours 24–48 for the 48-h timepoint), and cumulative-to-date values, representing total consumption from time zero through the stated timepoint. At the initial 24-h assessment these two constructs are mathematically equivalent, since no preceding interval exists to accumulate; all six opioid-reporting trials therefore contributed to a single pooled 24-h estimate. Beyond 24 h, only trials reporting the same construct at a given timepoint were pooled together. At 48 h and 72 h, four trials reported per-interval consumption [7, 17, 20, 21] and were pooled; Ho [19] reported cumulative-to-date consumption at 48 h and Li [15] reported cumulative-to-date consumption at 72 h, each contributing a single-study value that is presented descriptively rather than pooled (see Results). At 7 days, two trials reporting per-interval consumption were pooled [7, 21]; [15] cumulative 7-day value is likewise presented descriptively. Following standardization to a common unit and analytic construct, opioid consumption was pooled as a Mean Difference (MD) in MME with 95% CI within each construct-homogeneous timepoint, permitting clinically interpretable quantification of the opioid-sparing effect. Dichotomous data were represented by the risk ratio (RR). This metric offers a direct and clinically intuitive comparison of complication incidence between groups. Heterogeneity was assessed using the Chi-square test and the I^2^ value, with significance set at *P* < 0.05 and I^2^ > 50%. A random-effects model was applied in cases of significant heterogeneity; otherwise, a fixed-effects model was used. The DerSimonian–Laird (DL) method was used to estimate between-study variance (τ^2^) in all random-effects models, consistent with the default implementation in RevMan 5.4. This estimator was selected for its computational transparency, widespread adoption in the meta-analysis literature, and consistency with prior systematic reviews of perioperative duloxetine in arthroplasty. The Hartung–Knapp–Sidik–Jonkman (HKSJ) correction was not applied to confidence intervals; recent meta-epidemiological evidence specific to orthopaedic meta-analyses demonstrates that the DL estimator without HKSJ adjustment produces narrower confidence intervals and an elevated risk of false-positive results—an effect amplified in the presence of high heterogeneity—and this limitation should be considered when interpreting outcomes where I^2^ is substantial [23]. Beyond the opioid analyses, this same concern applies to three additional outcomes pooled under substantial heterogeneity: 24-h ambulatory pain (I^2^ = 74%) and ≥3-month pain, both rest and ambulatory (I^2^ = 70% and 69%, respectively). We therefore applied the Hartung–Knapp–Sidik–Jonkman adjustment to these three estimates as a post hoc sensitivity analysis, using the metafor package in R (test = “knha”), with results reported alongside the primary estimates. The nausea/vomiting and fatigue outcomes (I^2^ = 0% for each) were pooled under a fixed-effects model, to which this correction does not apply; their robustness is instead addressed via the leave-one-out sensitivity analysis reported below. For opioid consumption outcomes, where I^2^ ranged from 97 to 100% across all four timepoints, pooled estimates were retained rather than replaced by narrative synthesis: at this level of heterogeneity, a random-effects model estimates the mean of a distribution of true effects across diverse clinical settings rather than a single common effect, and pooling was retained to characterize the direction of the opioid-sparing signal across heterogeneous perioperative contexts. These opioid estimates should accordingly be treated as exploratory rather than as precise, generalizable effect sizes. We performed sensitivity analyses, systematically removing each study to evaluate result stability. For outcomes with ≥10 contributing studies, publication bias was assessed in R Statistical Software using the metafor package: visual inspection of the funnel plot, Egger’s weighted regression test for funnel plot asymmetry (regtest() function), trim-and-fill analysis (Duval and Tweedie method), and, for binary outcomes analysed under a fixed-effects model, Peters’ test as a supplementary asymmetry test. The Rosenthal Fail-Safe N was likewise calculated in R using metafor, with a robustness threshold set at 5 k + 10. Outcomes with fewer than 10 contributing studies were not assessed for publication bias, consistent with Cochrane Handbook recommendations. Statistical significance was defined as *P* < 0.05.

## Results

### Search results and characteristics of included studies

**Fig. 1:**
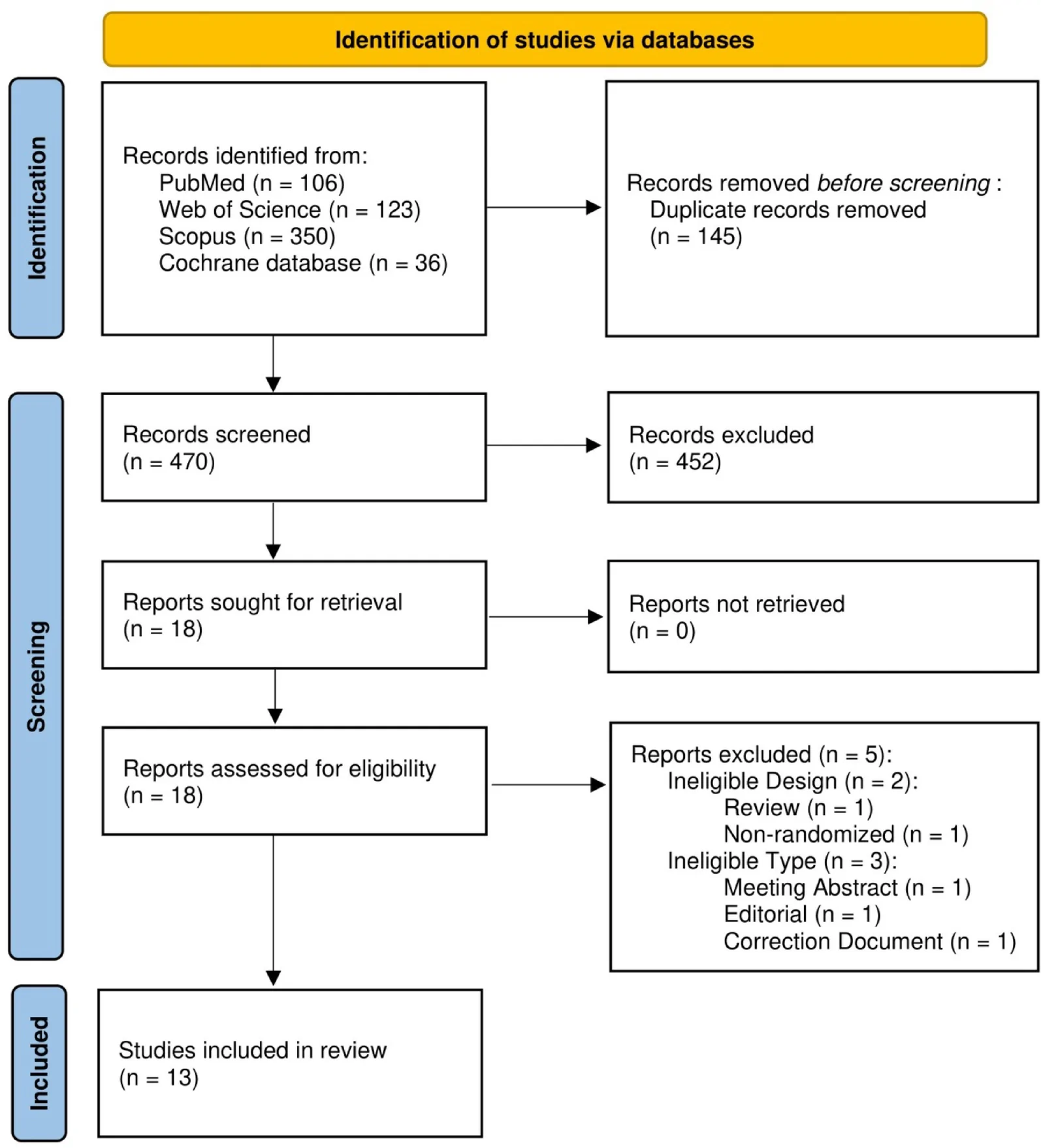
Preferred reporting items for systematic reviews and meta-analyses (PRISMA) flowchart of included studies

A total of 615 studies were identified from electronic databases. After removing duplicate references, 470 studies remained. Title and abstract screening excluded 452 records for irrelevance. Full-text review eliminated a further five. Finally, thirteen RCTs published between 2010 and 2024, including 1176 patients, met the inclusion criteria and were included in this meta-analysis. Baseline characteristics of included studies are demonstrated in Table 1. The study selection process is shown in Fig. 1. The mean age and sex distribution in studied groups are shown in Table 2.

**Table 2:** Patients’ characteristics

Author, year	Age (years)						Sex					
	Dulox group		Placebo group		Total		Dulox group		Placebo group		Total	
	mean	SD	mean	SD	mean	SD	Females	Males	Females	Males	Females	Males
Ho [19]	65.2	NR	65.7	NR	65	NR	16	7	17	7	33	14
YaDeau [20]	67	NR	63	NR	65	NR	28	25	26	27	54	52
Koh [13]	69.1	5.8	68.6	9.5	68.9	7.8	35	5	34	6	69	11
Kim [14]	71.2	6.5	67	7.1	69	7.1	17	2	16	4	33	6
Li [15]	52.7	12	50.2	13.2	51.5	12.6	26	22	24	24	50	46
Rienstra [11]	61.5	8.1	64	8.7	62.7	8.5	38	19	31	23	69	42
Yuan [7]	67.8	10.12	66.2	9.83	67	9.96	30	20	27	23	57	43
YaDeau [21]	63	11	64	7	63.5	9.2	40	40	35	45	75	85
Ding [12]	58	NR	61	NR	59.5	NR	21	13	18	15	39	28
Imani [10]^a^	67.6	4.3	65.5	6.4	66.55	5.49	19	1	20	0	39	1
Inamullah [16]	NR	NR	NR	NR	56.19	14.27	NR	NR	NR	NR	NR	NR
Pinsornsak [17]	65.1	7.2	66.9	6.4	66	6.8	36	6	36	6	72	12
Rajani [18]	71.64	7.01	70.98	7.63	71.31	7.3	30	23	28	25	58	48

SD, standard deviation; NR, not reported; Dulox, duloxetine

^a^See Table 1 footnote a for this trial’s three-arm design and arm-extraction rationale

### Quality assessment

**Fig. 2:**
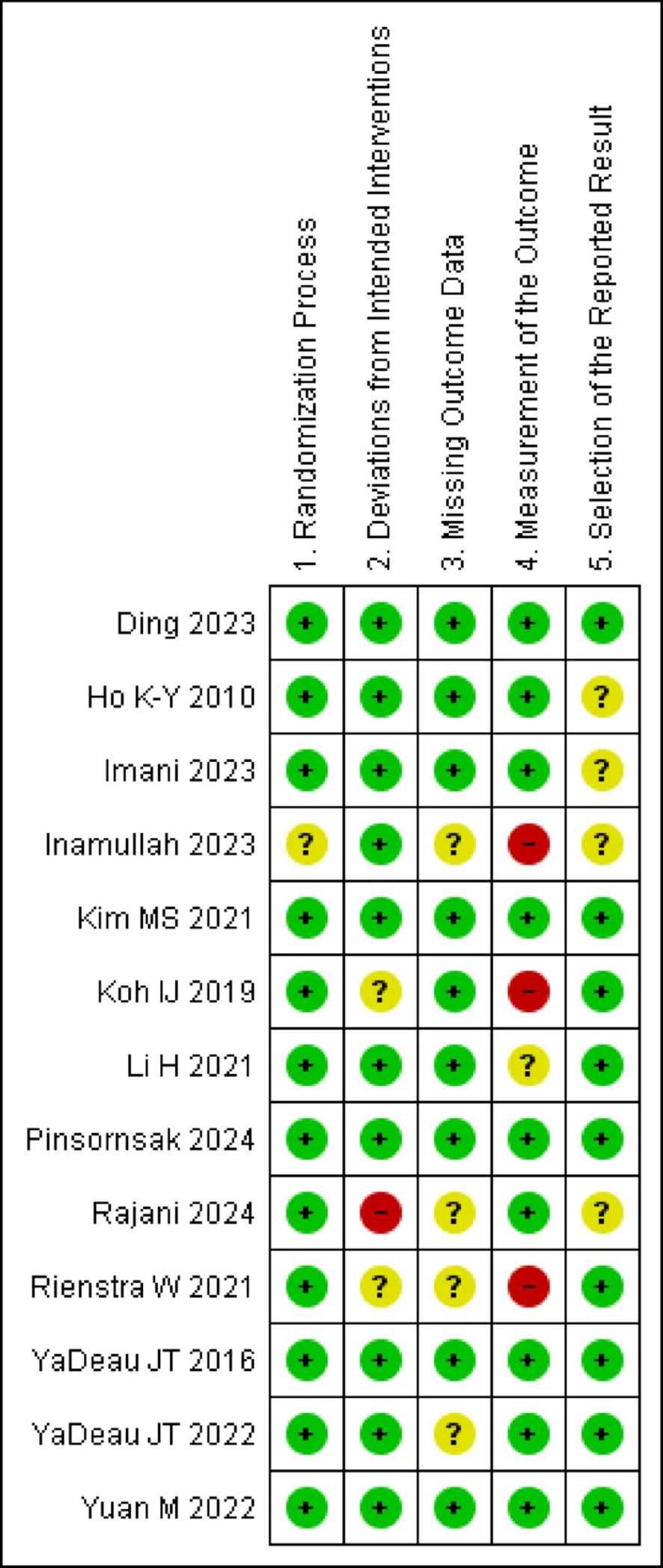
Risk of bias summary

**Fig. 3:**
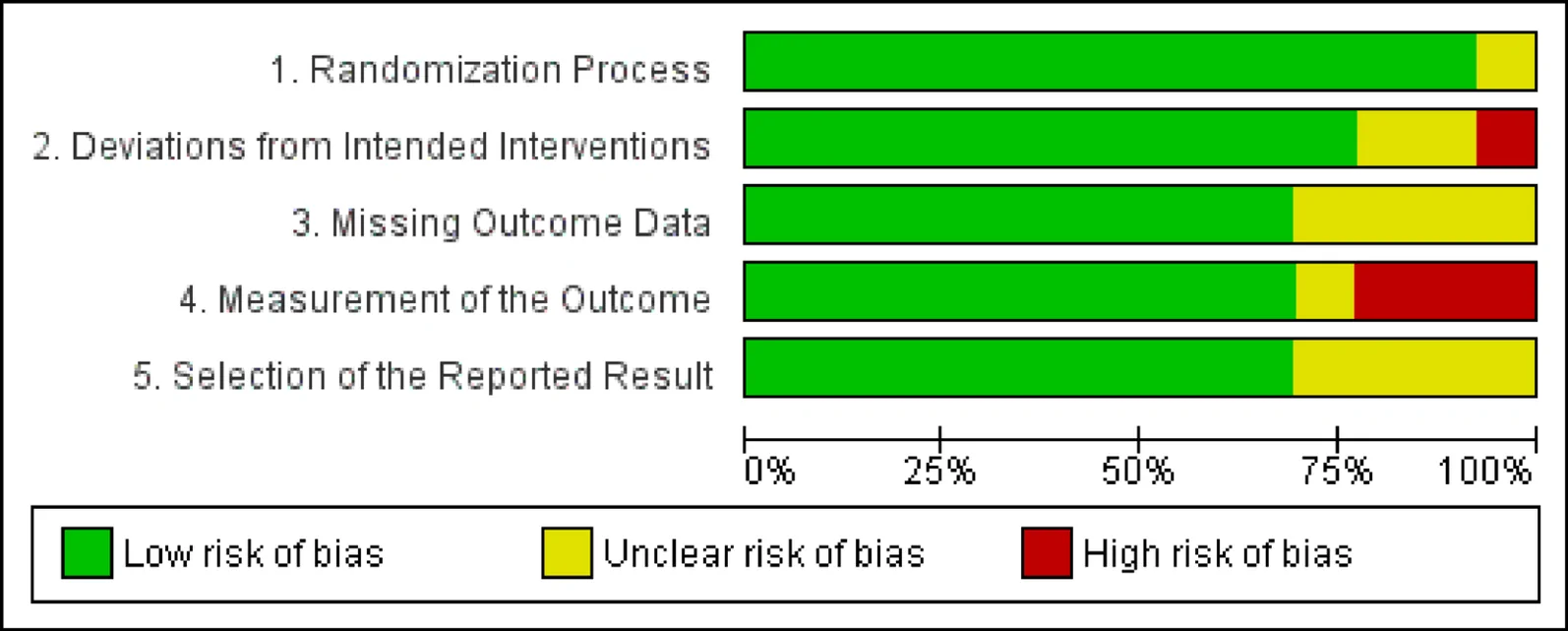
Risk of bias graph

The methodological quality of the 13 included trials was assessed using the RoB 2 tool, revealing a generally sound foundation with specific areas of concern in blinding and attrition. Figure 2 shows risk of bias summary of the enrolled studies. Figure 3 shows risk of bias graph of the enrolled studies. Randomization processes were adequate in 12 trials (92%), though Inamullah et al. [16] presented some concerns due to insufficient detail regarding allocation concealment.

A critical vulnerability was identified in outcome measurement for two studies: Rienstra et al. [11] and Inamullah et al. [16] were judged to be at high risk of bias because their open-label designs combined with subjective, patient-reported outcomes (e.g., VAS scores) created a high potential for observer and performance bias. Similarly, deviations from intended interventions led to a high risk of bias in the study by Rajani et al. [18], which failed to perform a true intention-to-treat analysis by excluding 7% of randomized participants for non-compliance.

Attrition and missing outcome data introduced some concerns in four trials; notably, Rienstra et al. [11] reported a 21% discontinuation rate in the intervention group due to adverse effects, potentially leading to data that were “missing not at random”.

Finally, four studies [10, 16, 18, 19] raised concerns regarding the selection of the reported result, primarily because they lacked a pre-registered protocol or a detailed statistical analysis plan to verify that the reported outcomes were not selectively chosen.

### Primary outcomes

#### VAS scores at rest and upon ambulation

##### VAS scores 24 h after surgery

**Fig. 4:**
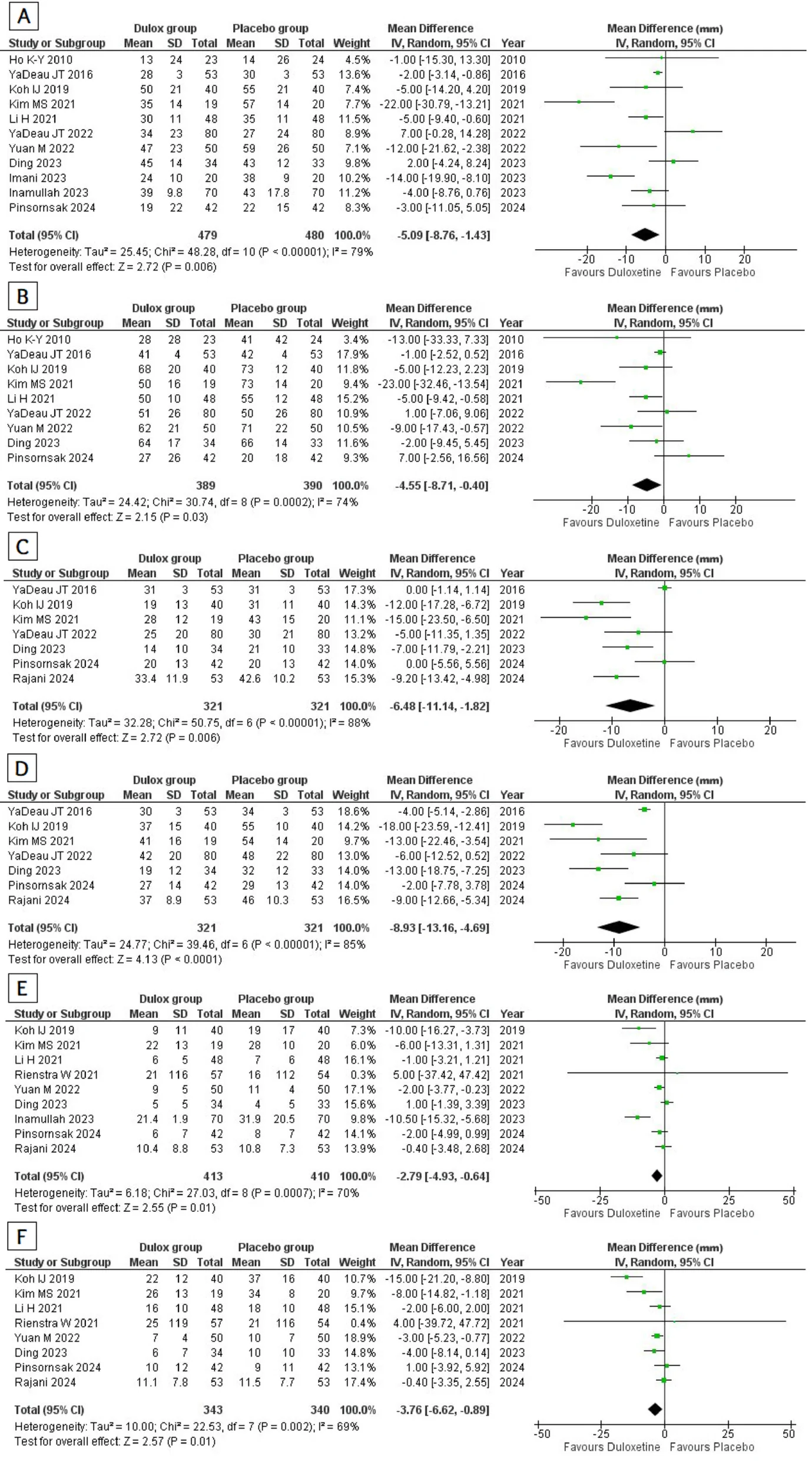
rVAS and aVAS pain scores. **A** rVAS scores 24 h after surgery; **B** aVAS scores 24 h after surgery; **C** rVAS scores 2 weeks after surgery; **D** aVAS scores 2 weeks after surgery; **E** rVAS scores≥ 3 months after surgery; **F** aVAS scores≥ 3 months after surgery. rVAS: visual analog score at rest, aVAS: visual analog score upon ambulation

Eleven studies (n = 959) reported rVAS scores and nine studies (n = 779) reported aVAS scores 24 h after surgery. A random-effects model was used due to significant heterogeneity. Compared to the placebo group, duloxetine significantly reduced pain at rest (MD −5.09 mm, 95% CI −8.76 to −1.43, *P* = 0.006, I^2^ = 79%) and pain upon ambulation (MD −4.55 mm, 95% CI −8.71 to −0.40, *P* = 0.03, I^2^ = 74%). Both reductions fall below the commonly accepted minimal clinically important difference (MCID) of 10 mm on a 0–100 mm VAS scale [24]. Given the substantial heterogeneity underlying the ambulatory pain estimate (I^2^ = 74%), an HKSJ-adjusted sensitivity analysis produced a wider 95% CI of −10.62 to 1.52 mm (*P* = 0.12); under this correction the effect no longer reached statistical significance. Details are shown in Fig. 4A and B.

##### VAS scores two weeks after surgery

Seven studies (n = 642) reported both rVAS and aVAS scores two weeks after surgery. Compared to placebo, duloxetine significantly decreased rVAS scores (MD −6.48 mm, 95% CI −11.14 to −1.82, *P* = 0.006, I^2^ = 88%) and aVAS scores (MD −8.93 mm, 95% CI −13.16 to −4.69, *P* < 0.0001, I^2^ = 85%). Neither reduction meets the 10 mm MCID threshold on a 0–100 mm scale [24]; the 2-week aVAS reduction approaches but does not exceed this threshold. Details are shown in Fig. 4C and D.

##### VAS scores three months or longer after surgery

Nine RCTs (n = 823) assessed rVAS scores and eight RCTs (n = 683) assessed aVAS scores ≥ 3 months after surgery. Compared to the placebo group, duloxetine significantly reduced both rVAS scores (MD −2.79 mm, 95% CI −4.93 to −0.64, *P* = 0.01, I^2^ = 70%) and aVAS scores (MD −3.76 mm, 95% CI −6.62 to −0.89, *P* = 0.01, I^2^ = 69%). Both reductions fall well below the 10 mm MCID threshold on a 0–100 mm scale [24]; the rVAS estimate was additionally sensitive to the exclusion of a single high-risk study (see Sensitivity analysis). Given the substantial heterogeneity underlying both estimates (I^2^ = 69–70%), HKSJ-adjusted sensitivity analysis produced a 95% CI of −5.85 to 0.28 mm (*P* = 0.069) for rVAS and −7.85 to 0.34 mm (*P* = 0.067) for aVAS; neither estimate retained statistical significance under this adjustment. This converges with the rVAS estimate’s already-documented sensitivity to single-study exclusion (see Sensitivity analysis, above)—two independent checks pointing to the same fragility. Details are shown in Fig. 4E and F.

**Fig. 5:**
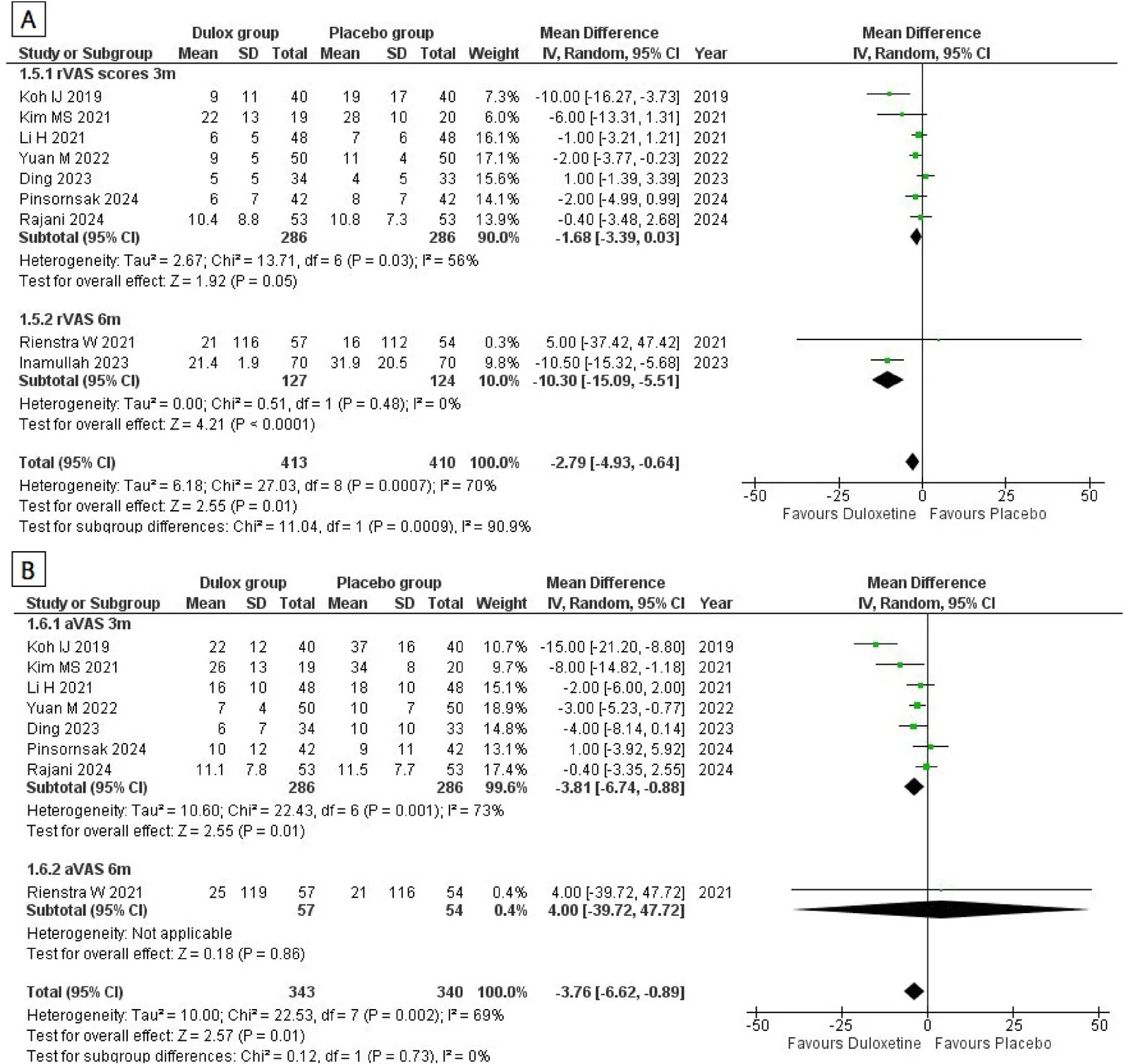
Subgroup analysis of late postoperative pain scores. **A** rVAS scores at 3 months and 6 months; **B** aVAS scores at 3 months and 6 months

A temporal subgroup analysis stratified the contributing studies by their exact reported follow-up timepoint to examine whether the ≥3-month effect differed across measurement windows. For rVAS, seven studies contributed data at the 3-month timepoint (n = 572) [7, 12–15, 17, 18] and two studies at the 6-month timepoint (n = 251) [11, 16]. At 3 months, the pooled rVAS effect was MD −1.68 mm (95% CI −3.39 to 0.03, *P* = 0.05, I^2^ = 56%); as the upper confidence limit crosses zero and *P* = 0.05 does not meet the pre-specified significance criterion of *P* < 0.05, this result is borderline non-significant and should not be described as a statistically significant reduction. At 6 months, the pooled rVAS effect was MD −10.30 mm (95% CI −15.09 to −5.51, *P* < 0.0001, I^2^ = 0%), the only VAS estimate in the present analysis to meet the 10 mm MCID threshold [24]. The subgroup difference between the 3-month and 6-month rVAS strata was statistically significant (Chi^2^ = 11.04, df = 1, *P* = 0.0009; I^2^ for subgroup differences = 90.9%), confirming marked temporal heterogeneity within the ≥3-month interval. For aVAS, seven studies reported data at 3 months (n = 572), yielding a significant pooled effect of MD −3.81 mm (95% CI −6.74 to −0.88, *P* = 0.01, I^2^ = 73%), which falls below the 10 mm MCID threshold [24]. Only one study (n = 111) [11] reported aVAS at 6 months (MD +4.00 mm, 95% CI −39.72 to +47.72, *P* = 0.86); as this is a single-study observation rather than a pooled estimate, no subgroup conclusion can be drawn for aVAS at 6 months. No statistically significant subgroup difference was detected for aVAS (Chi^2^ = 0.12, df = 1, *P* = 0.73). Details are shown in Fig. 5.

### Secondary outcomes

#### Opioid consumption 24 h after surgery

**Fig. 6:**
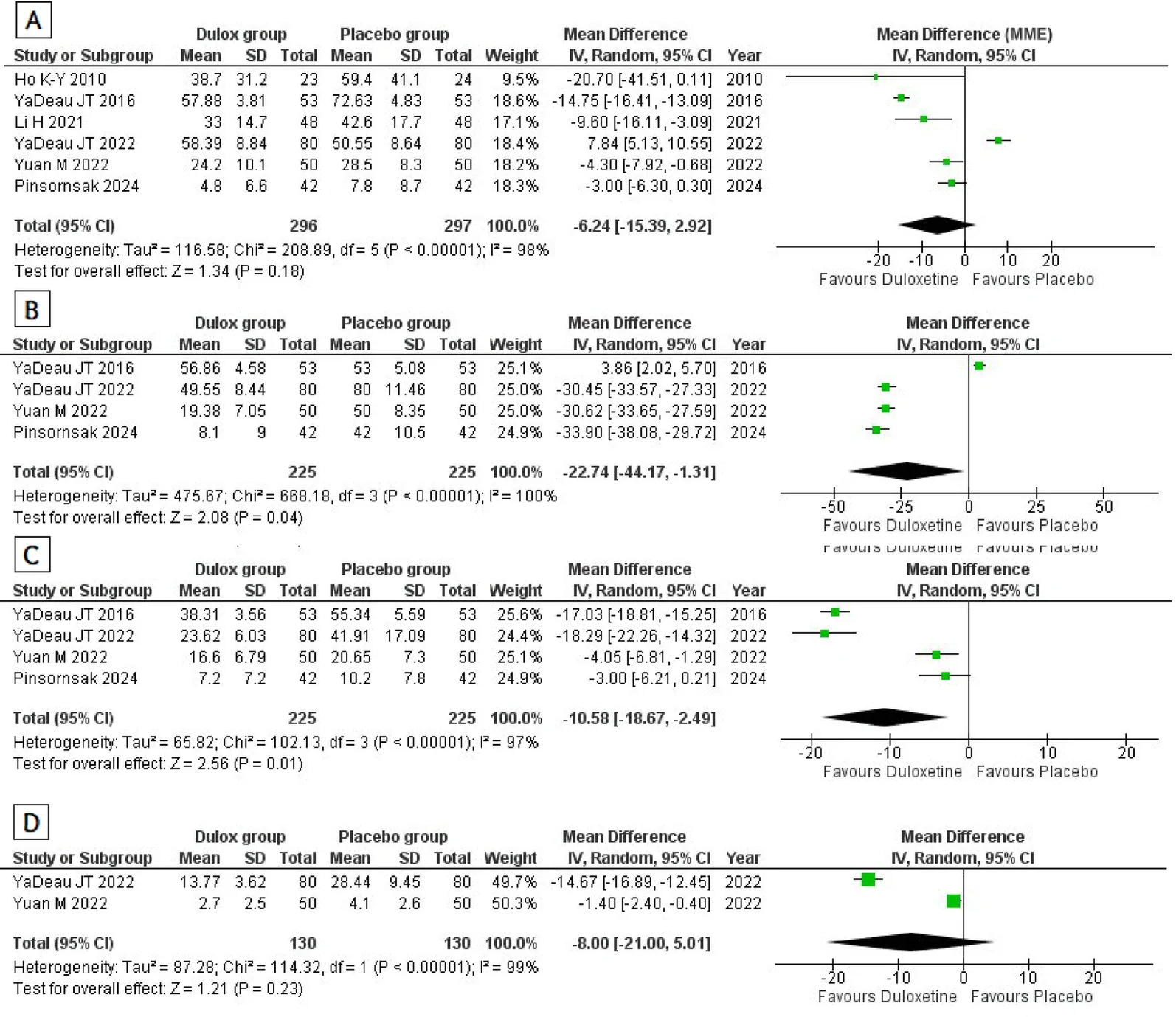
Opioid consumption (MME). Panel A pools per-interval and cumulative-to-date values as an equivalent construct at the initial 24-h timepoint; Panels B–D pool per-interval (daily) values only, with cumulative-to-date single-study values reported descriptively in the text (see Statistical Analysis and Results). **A** opioid consumption 24 h after surgery (n = 6 RCTs); **B** opioid consumption 48 h after surgery (n = 4 RCTs); **C** opioid consumption 72 h after surgery (n = 4 RCTs); **D** opioid consumption 7 days after surgery (n = 2 RCTs)

Opioid consumption at 24 h was reported in six RCTs (n = 593); at this initial timepoint, per-interval (daily) and cumulative-to-date values are mathematically equivalent (see Statistical Analysis), so all six trials contributed to a single pooled construct. Compared to placebo, duloxetine did not significantly reduce opioid consumption at 24 h (MD −6.24 MME, 95% CI −15.39 to 2.92, *P* = 0.18, I^2^ = 98%) (Fig. 6A). The extreme heterogeneity (I^2^ = 98%) indicates that the contributing studies are not estimating a common underlying effect; this pooled value characterizes the direction of effect across heterogeneous settings and should be interpreted as exploratory.

#### Opioid Consumption 48 h after surgery

Four RCTs reporting per-interval (daily) opioid consumption at 48 h were pooled [7, 17, 20, 21] (n = 450). After conversion to MME, duloxetine significantly reduced opioid consumption at this timepoint (MD −22.74 MME, 95% CI −44.17 to −1.31, *P* = 0.04, I^2^ = 100%) (Fig. 6B). The extremely wide confidence interval and total heterogeneity (I^2^ = 100%) indicate that the four contributing studies are not estimating a common effect, and this result should be interpreted as a fragile, borderline-significant signal rather than a precise estimate. Ho [19] additionally reported cumulative-to-date opioid consumption through 48 h (59.4 ± 43.5 vs 90.9 ± 54.3 MME); because this reflects a different construct from the per-interval values above (see Statistical Analysis), it is reported here descriptively rather than pooled.

#### Opioid Consumption 72 h after surgery

Four RCTs reporting per-interval (daily) opioid consumption at 72 h were pooled [7, 17, 20, 21] (n = 450). Duloxetine significantly reduced opioid consumption compared to placebo (MD −10.58 MME, 95% CI −18.67 to −2.49, *P* = 0.01, I^2^ = 97%) (Fig. 6C). This reduction of approximately 10.6 MME is closely consistent in magnitude with the estimate obtained before construct separation (MD −10.62 MME), and represents a clinically meaningful absolute decrease; however, the extreme between-study heterogeneity (I^2^ = 97%) limits the precision of this estimate, and it should be interpreted as a directional signal rather than a definitive effect size. Li [15] additionally reported cumulative-to-date opioid consumption through 72 h (50.4 ± 18.3 vs 61.2 ± 29.4 MME); because this reflects a different construct from the per-interval values above (see Statistical Analysis), it is reported here descriptively rather than pooled.

#### Opioid consumption 7 days after surgery

Two RCTs reporting per-interval (daily) opioid consumption at 7 days were pooled [7, 21] (n = 260). The reduction in opioid consumption with duloxetine did not reach statistical significance at this interval (MD −8.00 MME, 95% CI −21.00 to 5.01, *P* = 0.23, I^2^ = 99%) (Fig. 6D); with only two contributing trials, this heterogeneity estimate should be interpreted with particular caution. Li [15] additionally reported cumulative-to-date opioid consumption through 7 days (56.1 ± 21.9 vs 69.9 ± 40.8 MME); because this reflects a different construct from the per-interval values above (see Statistical Analysis), it is reported here descriptively rather than pooled.

### Adverse effects

**Fig. 7:**
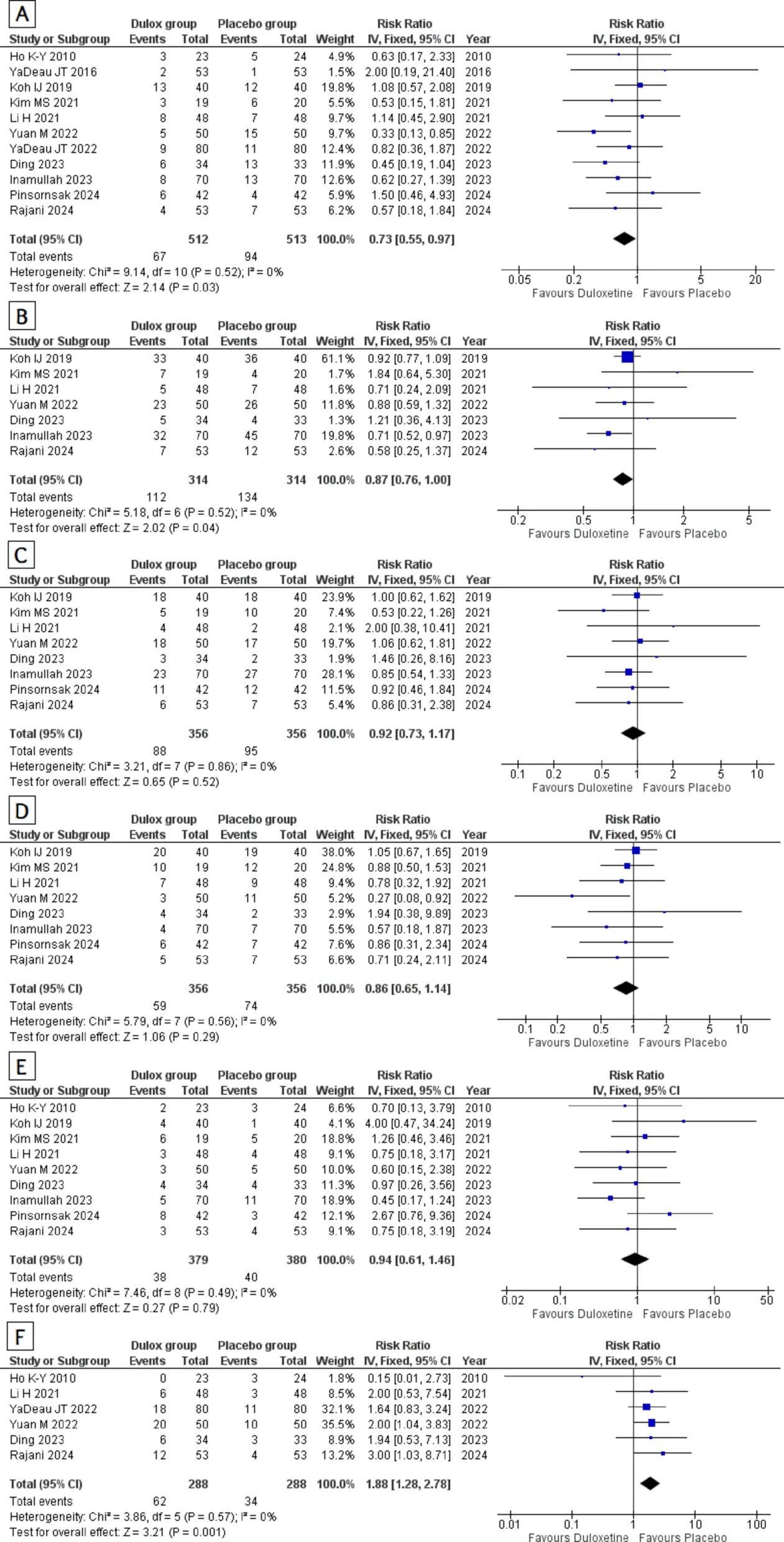
Adverse effects. **A** incidence of nausea/vomiting; **B** incidence of fatigue; **C** incidence of dry mouth; **D** incidence of constipation; **E** incidence of dizziness; **F** incidence of drowsiness/somnolence

Eleven studies reported adverse effects after surgery. Compared to the placebo, there were no significant differences in the incidence of dry mouth (RR 0.92, 95% CI (0.73, 1.17), *P* = 0.52, I^2^ = 0%) or constipation (RR 0.86, 95% CI (0.65, 1.14), *P* = 0.29, I^2^ = 0%). Dizziness rates were similarly unaffected (RR 0.94, 95% CI (0.61, 1.46), *P* = 0.79, I^2^ = 0%) between the duloxetine and placebo groups (Fig. 7C–E).

However, the duloxetine group had a significantly lower risk of nausea/vomiting (RR 0.73, 95% CI (0.55, 0.97), *P* = 0.03, I^2^ = 0%) compared to the placebo group (Fig. 7A). A borderline reduction in fatigue was also observed (RR 0.87, 95% CI (0.76, 0.996), *P* = 0.04, I^2^ = 0%); the upper limit rounds to 1.00 at two decimal places, but the unrounded value lies just below unity, consistent with the significant *P* value. Dedicated sensitivity analyses excluding Rajani 2024 or Inamullah 2023 individually each rendered this estimate non-significant, and the nausea/vomiting estimate also became borderline upon these exclusions (see Sensitivity analysis) (Fig. 7B). Both findings should therefore be interpreted with caution. Both estimates were derived under a fixed-effects model (I^2^ = 0% for each; see Publication bias, above, where Peters’ test was selected specifically because it is recommended for fixed-effects binary outcomes), so the HKSJ correction—which adjusts the between-study variance term in a random-effects model—does not directly apply. Robustness for these two outcomes is instead evidenced by the leave-one-out results above, which rendered both non-significant upon exclusion of Inamullah 2023 alone. Conversely, the duloxetine group had a significantly higher risk of drowsiness/somnolence (RR 1.88, 95% CI (1.28, 2.78), *P* = 0.001, I^2^ = 0%) compared to the placebo group (Fig. 7F).

### Sensitivity analysis

Leave-one-out sensitivity analysis confirmed the stability of the meta-analysis for short-term pain outcomes: rVAS and aVAS estimates at 24 h and 2 weeks remained statistically significant and directionally consistent regardless of which individual study was excluded. Opioid consumption was sensitive to the inclusion of specific studies, with notable variation in both effect magnitude and statistical significance across all four timepoints. Long-term pain (rVAS ≥3 months) and borderline adverse event estimates (nausea/vomiting and fatigue) were also sensitive to the exclusion of individual high-risk studies, as described in the dedicated analyses below. Given that Rajani et al. (2024) enrolled patients undergoing single-setting bilateral TKA—a clinically distinct scenario from the unilateral arthroplasty comprising the majority of the evidence base, as meta-analytic evidence demonstrates that simultaneous bilateral procedures carry substantially higher rates of perioperative transfusion, neurological complications, and early mortality compared with unilateral surgery [25]—a dedicated sensitivity analysis was performed excluding this study from all outcomes to which it contributed (rVAS and aVAS at 2 weeks and ≥3 months; adverse effects). Exclusion of Rajani 2024 did not materially alter any pain estimate: all pain reductions remained statistically significant and directionally consistent with the primary analysis (rVAS 2w: MD −5.97 mm, 95% CI −10.98 to −0.97; aVAS 2w: MD −9.01 mm, 95% CI −14.24 to −3.78; rVAS ≥3 m: MD −3.27 mm, 95% CI −5.72 to −0.81; aVAS ≥3 m: MD −4.52 mm, 95% CI −7.85 to −1.19). Rajani 2024 did not contribute to 24-h VAS or any opioid consumption analyses; those estimates are therefore unaffected by this exclusion. Upon exclusion of Rajani 2024, the fatigue estimate lost statistical significance (RR 0.88, 95% CI 0.76–1.01) and the nausea/vomiting estimate became borderline (RR 0.74, 95% CI 0.55–1.00), while drowsiness/somnolence remained robustly significant (RR 1.76, 95% CI 1.16–2.66); these shifts are consistent with the already-borderline nature of both primary adverse event estimates and reinforce cautious interpretation of those findings. Given the methodological concerns associated with Inamullah et al. (2023)—an open-label trial with blinding undocumented (NR)—a dedicated sensitivity analysis was performed excluding this study from all outcomes to which it contributed (rVAS at 24 h; rVAS at ≥3 months; nausea/vomiting, fatigue, dry mouth, constipation, and dizziness). Inamullah 2023 did not contribute data to aVAS at any timepoint, to opioid consumption at any timepoint, or to drowsiness/somnolence; those estimates are unaffected by this exclusion. Exclusion of Inamullah 2023 rendered the rVAS ≥3 month estimate substantially attenuated and essentially non-significant (MD −1.63 mm, 95% CI −3.28 to 0.02), compared with MD −2.79 mm (95% CI −4.93 to −0.64) in the primary analysis; the upper confidence limit crossing zero indicates that this long-term benefit cannot be considered statistically established once this high-risk study is removed. The nausea/vomiting estimate became non-significant (RR 0.75, 95% CI 0.55–1.02), and the fatigue estimate also became non-significant (RR 0.91, 95% CI 0.78–1.06). In contrast, the rVAS at 24 h remained robustly significant and essentially unchanged (MD −5.28 mm, 95% CI −9.48 to −1.08), and findings for dry mouth, constipation, and dizziness were unaffected. Taken together with the Rajani 2024 sensitivity analysis, these results indicate that the rVAS ≥3 month estimate, the nausea/vomiting result, and the fatigue result are each sensitive to the exclusion of a single high-risk study and should be interpreted with explicit caution; the 24-h and 2-week pain outcomes, by contrast, are robust.

Detailed sensitivity analysis data are shown in Additional file 3: Tables S2–S4.

### Publication bias

Publication bias was assessed for the two outcomes meeting the prespecified ≥10 study threshold: rVAS at 24 h (k = 11) and nausea/vomiting incidence (k = 11).

**Fig. 8:**
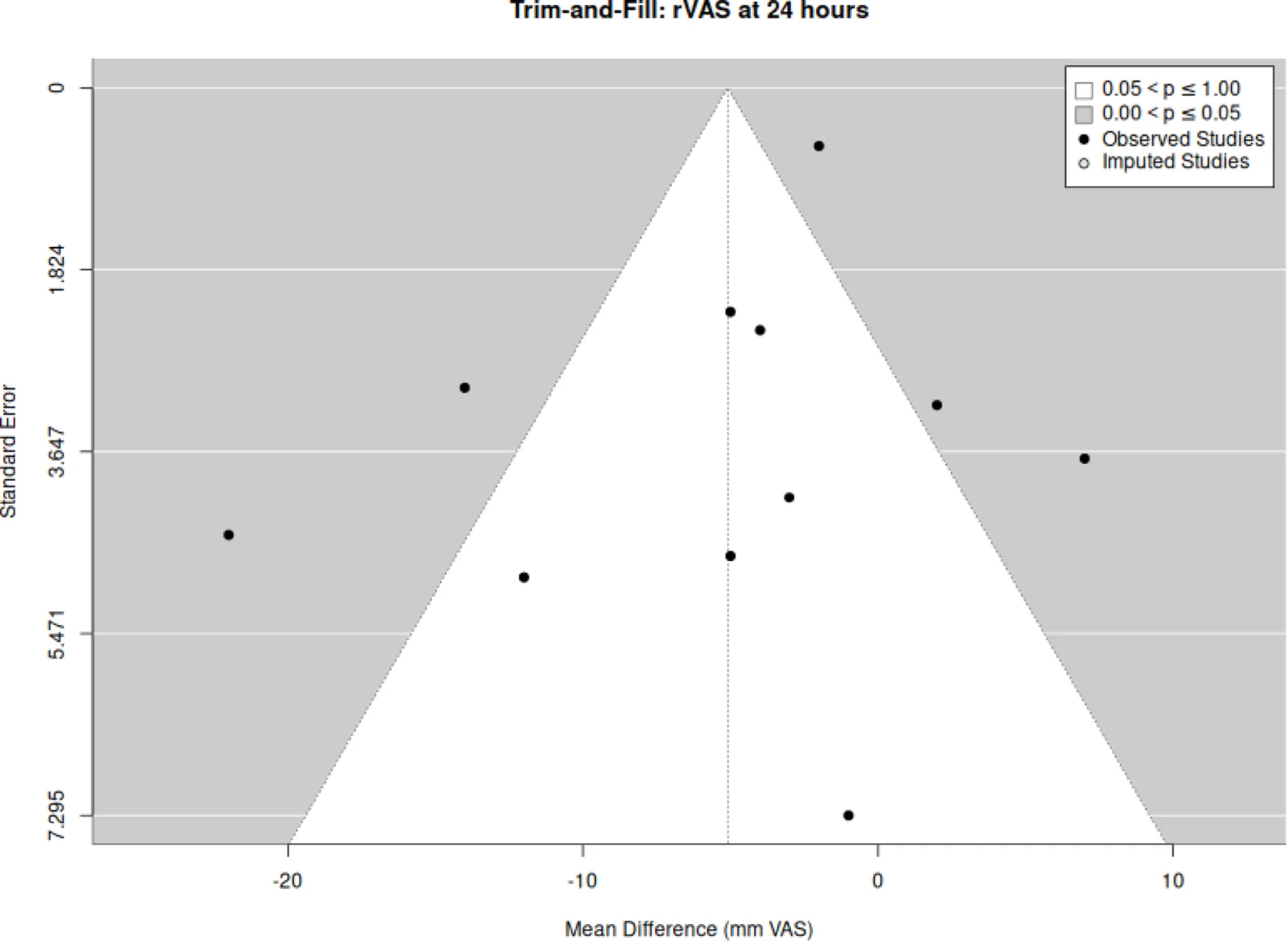
Trim-and-fill funnel plot for resting VAS pain (rVAS) at 24 h postoperatively (k = 11 studies)

For rVAS at 24 h, visual inspection of the trim-and-fill funnel plot (Fig. 8) revealed no marked asymmetry. Egger’s regression test was non-significant (t = −1.26, df = 9, *P* = 0.24), providing no statistical evidence of funnel plot asymmetry. Trim-and-fill analysis (Duval and Tweedie method) estimated zero missing studies on either side; the bias-adjusted pooled estimate was therefore identical to the observed estimate (MD −5.09 mm, 95% CI −8.76 to −1.43). The Rosenthal Fail-Safe N was 119—substantially exceeding the recommended robustness threshold of 65 (5 k + 10)—indicating that 119 unpublished null studies would be required to nullify this finding, and supporting its robustness.

**Fig. 9:**
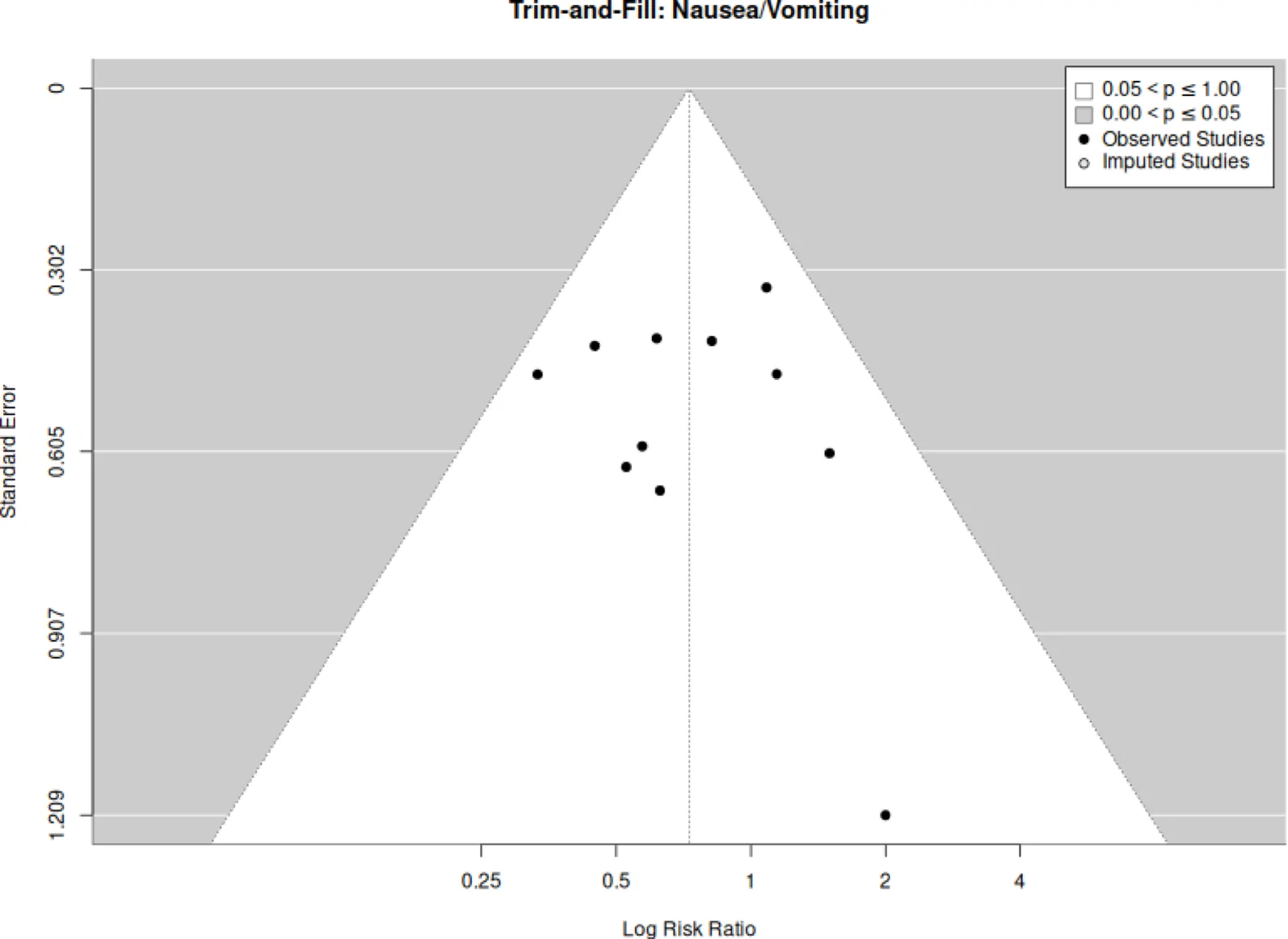
Trim-and-fill funnel plot for the incidence of nausea/vomiting (k = 11 studies)

For nausea/vomiting incidence, visual inspection of the funnel plot (Fig. 9) showed no notable asymmetry. Both Egger’s test (t = 0.13, df = 9, *P* = 0.90) and Peters’ test—recommended for binary outcomes analysed under a fixed-effects model [26]—were non-significant (*P* = 0.63), providing no statistical evidence of funnel plot asymmetry by either method. Trim-and-fill analysis estimated zero missing studies, and the bias-adjusted estimate remained unchanged. However, the Rosenthal Fail-Safe N was 6, falling below the robustness threshold of 65; while no asymmetric pattern was detectable by any applied test, the low Fail-Safe N indicates that as few as six unpublished null studies would render this result non-significant—a reflection of the finding’s borderline *p* value (*P* = 0.032)—and this result therefore warrants more cautious interpretation than the rVAS outcome.

Publication bias was not assessed for outcomes with fewer than 10 contributing studies, consistent with Cochrane Handbook guidance.

## Discussion

### Summary of main findings

This systematic review and meta-analysis of 13 randomized controlled trials involving 1176 patients synthesizes evidence on the perioperative use of duloxetine for pain management in total hip and knee arthroplasty. Our findings demonstrate that duloxetine, when added to standard multimodal analgesia, confers specific clinical benefits: Compared to placebo, duloxetine produced statistically significant reductions in pain at rest and during ambulation at all time points assessed—24 h (rVAS MD −5.09 mm; aVAS MD −4.55 mm), 2 weeks (rVAS MD −6.48 mm; aVAS MD −8.93 mm), and ≥3 months (rVAS MD −2.79 mm; aVAS MD −3.76 mm). A temporal subgroup analysis within this interval identified differential patterns at the 3-month and 6-month timepoints. However, all pain reductions fell below the accepted MCID of 10 mm on a 0–100 mm scale [24], indicating that the observed effects are statistically significant but of uncertain clinical magnitude. When opioid consumption data were standardized to MME and pooled as Mean Differences, statistically significant opioid-sparing effects were observed at 48 h (MD −22.74 MME, 95% CI −44.17 to −1.31, *P* = 0.04) and 72 h (MD −10.58 MME, 95% CI −18.67 to −2.49, *P* = 0.01); results at 24 h and 7 days did not reach statistical significance. All four timepoints were characterized by extreme statistical heterogeneity (I^2^ = 97–100%), severely limiting the precision and generalizability of the pooled opioid estimates. Regarding safety, duloxetine significantly reduced the risk of nausea/vomiting (RR 0.73) and significantly increased drowsiness/somnolence (RR 1.88). A borderline reduction in fatigue was also observed (RR 0.87, 95% CI 0.76–0.996), though an upper limit just below unity and sensitivity to individual study exclusion preclude a confident conclusion for this outcome. No significant differences were observed for dry mouth, constipation, or dizziness.

### Agreement with existing literature

Our findings are consistent with the broader literature in identifying duloxetine as a potential adjunct in perioperative pain management for joint arthroplasty. A directional opioid-sparing effect recurs across multiple reviews, although the specific timepoints reaching statistical significance vary between analyses; in the present analysis, point estimates favored duloxetine at all four opioid timepoints, but significance was reached only at 48 and 72 h. Critically, extreme between-study heterogeneity (I^2^ = 97–100%) affected all four timepoints, including the two that reached significance, so the 48- and 72-h findings should not be regarded as more secure than the null 24-h and 7-day estimates. Branton and colleagues’ 2021 meta-analysis of elective orthopedic surgery [27] first established this effect, demonstrating significant reductions in opioid consumption at 24 and 48 h postoperatively. Subsequent arthroplasty-specific meta-analyses by Azimi et al. [28]—as well as those by Zhong, Zhou, and Lin [9, 29, 30]—corroborated these findings, and collectively point to a consistent reduction in opioid requirements. Taken together, these analyses converge on the direction of an opioid-sparing effect, but—as discussed further below (see Temporal Patterns in Opioid Reduction)—the specific timepoints reaching significance differ across reviews, a pattern more consistent with substantial underlying heterogeneity than with a precise, reproducible pharmacokinetic time-course.

We found similar consistency in the reviewers’ conclusions for our pain outcomes. Meta-analysis showed that patients had statistically significantly lower pain scores in the first 2–3 weeks after surgery, which was aligned with the reviews by Yang et al. [31], Zhang et al. [32], Zhou et al. [30] and Lin et al. [9].

The secondary outcome of our study was safety. When pooling the results for our secondary outcomes, we found that the findings were consistent with prior reviews for the majority of adverse events. Evidence shows that drowsiness/somnolence is significantly increased with duloxetine use (RR 1.88). This adverse effect was also reported by Azimi et al. [28] (RR 1.87), Zhong et al. [29] and Lin et al. [9]. Additionally, duloxetine use was shown to decrease the incidence of nausea/vomiting (RR 0.73), which can be explained by duloxetine’s serotonin reuptake inhibition. The antiemetic properties of duloxetine, along with decreased opioid use, likely contributed to this finding. Evidence from Branton et al. [27], Zhong et al. [29] and Lin et al. [9] supports this conclusion. All other adverse events such as dry mouth, constipation and dizziness were not statistically significant in most reviews.

### Nuanced findings and points of divergence

#### Long-term pain outcomes

Our finding that duloxetine significantly reduced pain at ≥3 months postoperatively (both at rest and during ambulation) represents a point where our analysis diverges from several previous reviews. Yang et al. [31] found no significant difference in pain at 12 weeks, 6 months, or 12 months. Zhou et al. [30] reported no difference at 6 weeks or 3 months. Zhong et al. [29] found a significant reduction in ambulatory pain but not rest pain at ≥3 months. Most notably, the comprehensive review by Laigaard and colleagues [24], which examined all perioperative analgesic interventions for persistent pain after THA and TKA, found that while duloxetine significantly reduced movement pain at 3–24 months (mean difference −4.9 mm on a 100-mm scale), this effect fell below their predefined clinically important threshold of 10 mm. Our updated analysis yields a comparable long-term ambulatory pain reduction of MD −3.76 mm—similarly statistically significant yet below this same threshold—corroborating Laigaard’s conclusion that the long-term pain benefit of duloxetine does not reach clinical significance by conventional criteria.

Several factors may explain why our analysis detected significant long-term effects where others did not. First and most importantly, our meta-analysis includes the most recent trials, with literature searches extending through 2024, capturing studies by Ding et al. [12], Pinsornsak et al. [17], and Rajani et al. [18] that were not available to earlier reviewers. These newer trials may be contributing additional statistical power and evidence for long-term benefit. Second, by analyzing rest and ambulatory pain separately, we may have preserved sensitivity that other reviews lost by combining these distinct outcomes. Third, the small effect sizes we observed (MD −2.79 to −3.76 mm on a 0–100 mm scale) are consistent with the modest effects reported by others, and fall well below the conventional MCID of 10 mm; the difference lies in whether these effects reach statistical significance with an expanded sample.

To address the concern that the ≥3-month interval conflates biologically distinct measurement windows, a temporal subgroup analysis was performed, stratifying the contributing studies by their exact reported follow-up timepoint. At 3 months, the rVAS reduction (MD −1.68 mm, *P* = 0.05) was borderline non-significant—failing to reach the pre-specified significance criterion of *P* < 0.05—while the aVAS reduction (MD −3.81 mm, *P* = 0.01) remained significant; both fall well below the 10 mm MCID, consistent with the modest overall ≥3-month estimates. At 6 months, the rVAS subgroup yielded a substantially larger and strongly significant reduction (MD −10.30 mm, 95% CI −15.09 to −5.51, *P* < 0.0001, I^2^ = 0%)—the only long-term result meeting the 10 mm MCID threshold. However, this estimate rests on exactly two contributing studies, one of which is Inamullah [16]: the same open-label, high-risk study whose exclusion in the dedicated sensitivity analysis rendered the overall rVAS ≥3-month estimate non-significant. The strongly significant 6-month rVAS signal is therefore not independent of the study that already drives fragility in the overall ≥3-month estimate and must be interpreted with corresponding caution. For aVAS at 6 months, only a single study contributed data (Rienstra 2021; MD +4.00 mm, *P* = 0.86) [11], showing no directional effect; no pooled conclusion can be drawn from a single-study observation. Taken together, the temporal subgroup analysis reveals that the statistical significance of the overall ≥3-month rVAS estimate is disproportionately driven by the 6-month stratum, which is itself heavily dependent on a single high-risk study, while the 3-month subgroup—comprising seven studies with a more favorable risk profile—does not reach statistical significance independently. This convergence with the Inamullah 2023 sensitivity analysis (see Sensitivity analysis) reinforces the recommendation for cautious interpretation of the ≥3-month rVAS estimate.

#### Heterogeneity of enrolled patient populations

Three of the 13 included trials restricted enrollment to pharmacologically pre-selected populations. Koh et al. [13] and Kim et al. [14] enrolled exclusively patients meeting criteria for central sensitization—defined in Kim et al. by a Central Sensitization Inventory (CSI) score ≥40—while Ding et al. [12] enrolled only patients with documented depressive symptoms undergoing THA. Together, these three trials contributed 186 of 1176 participants (15.8%) to the pooled sample: Koh 2019 and Kim 2021 each contributed to all six VAS timepoint analyses and to five of the six adverse event analyses (all except drowsiness/somnolence); Ding 2023 contributed to all six VAS analyses and all six adverse event analyses.

This enrollment pattern has a direct implication for interpretation of the pooled estimates. Duloxetine’s analgesic mechanism—SNRI-mediated reinforcement of descending inhibitory pain pathways—is most directly relevant when those pathways are dysregulated, as in states of central sensitization; its antidepressant action represents a co-mechanism in patients with depressive comorbidity. The expected effect size in populations selected for these conditions therefore exceeds that achievable in unselected arthroplasty patients in whom neither central sensitization nor depressive symptoms are enrollment criteria. Pooling these three trials alongside the ten general-population cohorts introduces a systematic directional bias toward overestimating the treatment effect applicable to the average joint replacement candidate. Future meta-analyses should stratify contributing trials by patient phenotype—unselected versus centrally sensitized versus depression-screened—and future trials targeting the general arthroplasty population should consider pre-specifying enrollment criteria that exclude patients meeting established CSI thresholds, to permit an unconfounded estimate of duloxetine’s incremental contribution.

#### Temporal patterns in opioid reduction

Our opioid consumption analysis was able to detect a trend across time points that others were unable to detect due to our pooled sample size. Following standardization of opioid data to MME, statistically significant reductions were detected at 48 h (MD −22.74 MME, 95% CI −44.17 to −1.31, *P* = 0.04, I^2^ = 100%) and 72 h (MD −10.58 MME, 95% CI −18.67 to −2.49, *P* = 0.01, I^2^ = 97%), with non-significant point estimates at 24 h (MD −6.24 MME, *P* = 0.18) and 7 days (MD −8.00 MME, *P* = 0.23). The 48-h estimate, although the larger of the two significant findings in magnitude, carries an extremely wide confidence interval (− 44.17 to −1.31 MME) and total heterogeneity (I^2^ = 100%)—indicating that the four contributing studies are not estimating a common underlying effect, and this result should be read as a fragile, borderline-significant signal rather than a precise estimate. The 72-h finding—a reduction of approximately 10.6 MME—is comparatively more stable (I^2^ = 97%) and corresponds to a clinically meaningful absolute decrease, broadly consistent with duloxetine’s reported pharmacokinetic profile, in which analgesic effects become more consistently detectable after 48–72 h of administration; even so, this estimate remains subject to substantial heterogeneity and should be treated as a directional signal rather than a definitive effect size. Zhong et al. [29] and Azimi et al. [28] detected significant opioid-sparing effects across multiple earlier timepoints using different methodological approaches to data pooling, which may account for the divergent patterns of significance observed across analyses. Across all four timepoints in the present analysis, extreme heterogeneity (I^2^ = 97–100%) means that none of the pooled opioid estimates can be treated as a precise, generalizable effect size; they are best understood as hypothesis-generating, capturing the overall direction of an opioid-sparing signal across clinically diverse settings rather than providing a treatment effect applicable to any specific perioperative protocol.

### Novel contributions of the present meta-analysis

#### Incremental contribution relative to prior reviews

This meta-analysis, comprising 13 RCTs and 1176 patients, updates the evidence already synthesized by the closest prior reviews on this question—Jones [8], Lin [9], Azimi [28], and Zhong [29]—which pooled duloxetine against placebo in THA and TKA across most of the same trial base. Relative to these four reviews, the present analysis is not a new question but an incremental synthesis: it contributes five trials published between 2023 and 2024 that were not available to them [10, 12, 16–18], together with the timepoint-resolved temporal analysis and the sensitivity and quality-assessment framing detailed in the sections below.

#### Most comprehensive adverse event analysis

Our adverse event analysis, incorporating data from 11 studies, is the largest analysis of duloxetine’s safety in this surgical cohort to date. We found significantly reduced nausea/vomiting and a borderline reduction in fatigue. However, this was accompanied by significantly increased drowsiness/somnolence—a pairing of outcomes that clarifies the risk–benefit tradeoff not visible in smaller samples. These data allow more specific shared decision-making with patients.

#### Nuanced temporal analysis

By examining opioid consumption at four distinct time points (24 h, 48 h, 72 h, 7 d) and pain at three time points (24 h, 2wk, ≥3mo), we provide a clearer timeline of duloxetine’s effects. This mapping helps clinicians set patient expectations regarding the onset and duration of benefit.

#### Rigorous quality assessment and sensitivity analysis

Our use of the Cochrane RoB 2 tool, with detailed identification of specific methodological concerns in individual trials (e.g., open-label designs in Rienstra et al. [11] and Inamullah [16], lack of intention-to-treat analysis in Rajani [18]), provides a direct assessment of evidence quality. The sensitivity analysis showing instability in opioid consumption findings—an important qualification—represents a methodological approach that strengthens confidence in the robust data while appropriately flagging the tentative signals.

### Clinical implications

Evidence from our meta-analysis and the existing literature points to specific considerations for clinicians managing pain after THA and TKA.

First, duloxetine 60 mg daily—initiated preoperatively or on the day of surgery and continued for at least 2 weeks postoperatively—may be considered as a component of multimodal analgesia regimens for appropriately selected patients, with the strongest pharmacologic rationale in those with features of central sensitization or comorbid depressive symptoms, in whom duloxetine’s SNRI mechanism directly targets the underlying pain phenotype. Evidence supports a directional opioid-sparing effect, with statistical significance confirmed at 48 and 72 h postoperatively; the consistency of this effect across the broader perioperative period is limited by substantial heterogeneity. Duloxetine also significantly reduced the incidence of nausea/vomiting, which may benefit early recovery; however, this finding became non-significant on leave-one-out sensitivity analysis excluding a single high-risk trial (Inamullah 2023; RR 0.75, 95% CI 0.55–1.02) and should be regarded as a tentative rather than an established benefit.

Second, clinicians should counsel patients regarding the expected side effect profile. The increased risk of drowsiness/somnolence—approximately 1.8-fold—is predictable. Discuss this preoperatively, particularly with older adults at increased fall risk. Patients should be advised to exercise caution during the first postoperative week and to avoid driving or operating machinery if experiencing significant sedation. Conversely, patients can be reassured that duloxetine is unlikely to exacerbate constipation or dizziness. It may actually reduce nausea.

Third, the optimal timing and duration of therapy deserve consideration. We saw no significant opioid reduction at 24 h, whereas reductions reached significance at 48 and 72 h—though the 48-h estimate carries total heterogeneity (I^2^ = 100%) and should be interpreted cautiously. This pattern is broadly consistent with initiating duloxetine several days preoperatively, as many included trials did, so that therapeutic levels are established over the days 2–3 window; given the fragility of the 48-h estimate, however, this timing rationale is best read as a directional hypothesis rather than an established pharmacokinetic mechanism. Continuing therapy for at least 2 weeks captures the period of maximal opioid-sparing benefit; the decision to extend treatment beyond this point should be individualized based on persistent pain, side effect tolerance, and patient preference.

Fourth, the effect sizes for pain reduction are modest—particularly in the long term. Duloxetine is not a standalone solution. It is one component of a comprehensive multimodal strategy: regional anesthesia, non-opioid analgesics, and non-pharmacological interventions. The marked variation in background analgesic protocols across included trials (see Limitations: Variability in Background Analgesic Regimens) underscores that the reported effect estimates reflect specific analgesic contexts and may not transfer directly to protocols with substantially different co-interventions.

## Limitations

### Heterogeneity

Substantial statistical heterogeneity was present in many of our pooled analyses. This heterogeneity likely stems from clinical and methodological diversity among the included studies. Differences in duloxetine dosing (30 mg vs. 60 mg daily), timing of initiation (preoperative day 2 vs. day of surgery), and duration of therapy (2 weeks to 6 weeks) all likely contributed; the contribution of marked variability in background analgesic protocols is addressed in the dedicated subsection below. We employed random-effects models to account for this, but the presence of substantial heterogeneity means that pooled effect sizes should be interpreted as average effects across varying clinical contexts—not precise estimates applicable to all settings. Furthermore, the DerSimonian–Laird heterogeneity estimator was used without the Hartung–Knapp–Sidik–Jonkman correction; as demonstrated in a recent meta-epidemiological study of orthopaedic meta-analyses, this combination may yield artificially narrow confidence intervals and an elevated rate of false-positive results, with the effect most pronounced for outcomes with high I^2^ values—particularly the opioid consumption analyses reported here (I^2^ = 97–100%) [23]. The same concern extends to two further outcomes pooled under substantial heterogeneity: 24-h ambulatory pain (I^2^ = 74%) and ≥3-month pain (I^2^ = 69–70%). We addressed this directly with an HKSJ-adjusted sensitivity analysis for these three estimates (see Results); all three lost statistical significance under this more conservative adjustment. The nausea/vomiting and fatigue estimates (I^2^ = 0% each) were pooled under a fixed-effects model, to which HKSJ does not apply; both were nonetheless shown to be fragile via leave-one-out analysis (see Sensitivity Analysis and Risk of Bias, above), losing significance upon exclusion of Inamullah 2023 alone, and should be read with the same caution as the opioid estimates. At I^2^ values of 97–100%, the opioid consumption estimates represent the most heterogeneous findings in this analysis; they reflect the overall direction of the opioid-sparing signal across diverse clinical settings rather than a precise, generalizable treatment effect. Pooling was retained—rather than replaced by narrative synthesis—to characterize this directional signal, and these estimates should accordingly be treated as hypothesis-generating. Even the two statistically significant opioid results, at 48 h (I^2^ = 100%) and 72 h (I^2^ = 97%), should not be used as quantitative clinical benchmarks without corroboration from trials conducted under more homogeneous analgesic protocols — the 48-h estimate, given its total heterogeneity, warrants especially cautious interpretation.

### Variability in background analgesic regimens

The background analgesic protocols across the 13 included trials varied substantially in composition and complexity. Three trials [13, 14, 20] incorporated pregabalin as a routine co-intervention—a gabapentinoid that independently modulates central pain sensitization—while the remaining ten did not. Periarticular infiltration was used in six trials and absent or unreported in seven; dedicated regional nerve blocks (adductor canal block and IPACK) were employed in YaDeau [21], while Pinsornsak [17] used combined spinal and continuous epidural analgesia—a neuraxial technique not replicated in any other included study. Routine NSAID and paracetamol use was similarly inconsistent; at the opposite extreme, Ho and Li [15, 19] specified no background analgesic protocol beyond duloxetine versus placebo. This spectrum—from minimal background analgesia to dense multimodal co-intervention—is the most plausible primary driver of the extreme between-study heterogeneity (I^2^ = 74–99%) and means that the pooled duloxetine effect estimates cannot be assumed applicable to any specific clinical protocol. Study-level background regimens are summarized in Additional file 4: Table S5. Future trials should standardize or stratify background analgesic protocols to permit a more precise evaluation of duloxetine’s incremental contribution.

### Risk of bias in included studies

Our quality assessment (RoB 2) revealed methodological concerns even within this subset of RCTs. The open-label designs in Rienstra et al. [11] and Inamullah et al. [16] introduced potential performance and detection bias—particularly for subjective outcomes like pain. Inamullah et al. [16] raised additional concerns: blinding was undocumented (NR), limiting independent verifiability. Notwithstanding these concerns, the study was retained in the primary analysis in accordance with the pre-specified inclusion criteria (RCT design with extractable primary outcome data); a dedicated sensitivity analysis was performed to quantify its influence on pooled estimates (see Sensitivity analysis). Rajani et al. [18] did not perform an intention-to-treat analysis, excluding 7% of randomized participants; this raises specific concerns about attrition bias. Sensitivity analysis confirmed robustness of 24-h and 2-week pain estimates; however, the rVAS ≥3 month estimate and both borderline adverse event findings (nausea/vomiting and fatigue) were each rendered non-significant upon exclusion of Inamullah et al. [16] alone, confirming that this high-risk study exerts a disproportionate influence on those specific outcomes. Opioid consumption results were broadly sensitive to individual study exclusion across all timepoints. Readers should accordingly accord priority to the sensitivity-adjusted interpretation for long-term pain, nausea/vomiting, and fatigue outcomes. The Rosenthal Fail-Safe N for nausea/vomiting incidence was 6, indicating that this result—while supported by non-significant asymmetry tests and no imputed studies on trim-and-fill analysis—would be nullified by as few as six unpublished null studies and should therefore be interpreted with appropriate caution.

### Inability to perform subgroup analyses

Despite our large overall sample, we were unable to perform planned subgroup analyses based on duloxetine dose, treatment duration, or surgical procedure (THA vs. TKA), due to the limited number of studies per category. A temporal subgroup analysis of the ≥3-month VAS outcomes was performed, stratifying contributing studies into 3-month and 6-month strata. The inability to separate THA and TKA outcomes represents a more substantive constraint than a logistical one. The two procedures differ in their postoperative pain biology: TKA involves greater soft-tissue disruption and routine tourniquet application—tourniquet use has been demonstrated in randomized trial meta-analysis to produce significantly greater early postoperative knee and thigh pain compared with tourniquet-free surgery [33], a physiological burden that is simply absent in THA—and is associated with preoperative central sensitization whose adverse effects on postoperative pain outcomes are particularly pronounced in TKA compared with THA [34], as further detailed in the preceding Heterogeneity of Patient Populations subsection. These procedural differences translate into systematically distinct opioid requirements and duration of postoperative opioid use [35]. Furthermore, even within TKA, procedural variation in surgical technique—such as alignment strategy—produces statistically significant differences in early postoperative pain and functional scores [36], indicating that the TKA category is itself heterogeneous and that pooled estimates cannot be assumed to represent any single technical approach. The pooled estimates reported in this meta-analysis therefore cannot be confidently applied to either procedure individually; the true effect magnitude in a TKA cohort may differ materially from that in a THA cohort, and the current evidence base cannot quantify that difference. Future research should investigate whether 30 mg daily provides comparable efficacy to 60 mg—with potentially lower sedation risk—and whether extending therapy beyond 2 weeks confers additional long-term benefit. Dedicated single-procedure trials—or future meta-analyses restricted to TKA or THA cohorts respectively—are needed to generate procedure-specific effect estimates and determine whether the directional benefits observed in this pooled analysis are reproducible and of comparable magnitude in each arthroplasty type independently.

### Heterogeneity of patient populations

Three included trials enrolled pharmacologically pre-selected populations rather than general arthroplasty candidates: Koh et al. (2019) [13] and Kim et al. [14] restricted enrollment to centrally sensitized patients, and Ding et al. [12] enrolled exclusively patients with depressive symptoms, contributing a combined 186 of 1176 participants (15.8%) to the pooled estimates. As detailed in the preceding Discussion section, duloxetine’s mechanism directly targets the underlying pathology in both phenotypes, and the effect sizes observed in these trials may not be representative of those achievable in the general arthroplasty population. This clinical population heterogeneity is distinct from the statistical heterogeneity addressed in the first limitations subsection and is not resolvable by a random-effects model, which characterises the distribution of true effects across diverse settings without isolating the contribution of each patient subtype. Individual patient data (IPD) meta-analysis would be required to quantify the differential contribution of selected versus unselected populations to the pooled estimates reported here.

## Future research directions

The role of duloxetine for pain control after total joint arthroplasty requires further study. Head-to-head trials comparing various duloxetine dosing regimens (eg, 30 vs 60 mg daily dosing; shorter vs longer duration) with equivalence-powered trials would better delineate which regimen(s) offer the best efficacy-tolerability profile. Trials powered to detect a difference between THA and TKA cohorts would clarify whether patient or procedure-specific factors influence the analgesic effects of duloxetine. Trials with longer term follow-up (≥ 6 months) that employ standardized, validated outcome measures—including cross-culturally adapted instruments such as the recently validated Arabic KOOS-12 for non-English-speaking cohorts—would better establish duloxetine’s effect on outcomes associated with persistent pain and return of function [37]. Studies targeting specific subpopulations of patients (patients with evidence of centralized pain mechanisms prior to surgery, with anxiety/depression, or at risk for prolonged opioid use/failure to taper off opioids after surgery) may help to more narrowly define a role for duloxetine in the future.

## Conclusions

The results of this meta-analysis suggest that perioperative duloxetine, when added to multimodal analgesic regimens for total hip and knee arthroplasty, is associated with statistically significant reductions in postoperative pain across assessed timepoints and a significant opioid-sparing effect at 48 and 72 h postoperatively. Notably, the long-term (≥ 3-month) reduction in rest pain (rVAS) cannot be considered statistically established once a single high-risk trial (Inamullah 2023) is excluded (MD −1.63 mm, 95% CI −3.28 to 0.02, versus MD −2.79 mm, 95% CI −4.93 to −0.64, in the primary analysis). However, all pain reductions, expressed as mean differences on a 0–100 mm VAS scale, ranged from −2.79 to −8.93 mm and fell below the conventional minimal clinically important difference of 10 mm. Clinicians should therefore interpret the pain-reduction benefit as statistically real but of modest absolute magnitude. Furthermore, the substantial-to-extreme heterogeneity characterizing the pooled estimates—particularly for opioid consumption outcomes (I^2^ = 97–100%)—limits confidence in their precision and generalizability, and the findings should be treated as hypothesis-generating rather than definitive. Duloxetine is generally well tolerated in this setting but does lead to an increased risk of drowsiness/somnolence that should be communicated to patients. Taken together, the statistically significant but sub-MCID pain reductions, the opioid-sparing effect confirmed at 48 and 72 h, and the reduction in nausea/vomiting support duloxetine as a candidate adjunct within perioperative multimodal regimens for total joint arthroplasty, subject to confirmation in studies with more homogeneous populations and standardized background analgesia. As with the long-term rVAS finding above, however, the nausea/vomiting reduction becomes non-significant on exclusion of the same high-risk trial (Inamullah 2023; RR 0.75, 95% CI 0.55–1.02) and should be interpreted with corresponding caution.

## Supplementary Information

Below is the link to the electronic supplementary material.


Supplementary Material 1



Supplementary Material 2



Supplementary Material 3



Supplementary Material 4


## Data Availability

The datasets used and/or analysed during the current study are available from the corresponding author on reasonable request.

## Abbreviations

aVAS: Visual analog score upon ambulation
CI: Confidence interval
MMA: Multimodal analgesia
NR: Not reported
PICO: Population, intervention, comparison, outcome
PRISMA: Preferred reporting items for systematic reviews and meta-analyses
PROSPERO: International prospective register of systematic reviews
RCT: Randomized controlled trial
RoB: Risk of bias
RR: Risk ratio
rVAS: Visual analog score at rest
SD: Standard deviation
SNRI: Selective serotonin-norepinephrine reuptake inhibitor
THA: Total hip arthroplasty
TKA: Total knee arthroplasty
VAS: Visual analog scale

## References

[1] Colomina J, Drudis R, Torra M, Pallisó F, Massip M, Vargiu E, et al. Implementing mHealth-enabled integrated care for complex chronic patients with osteoarthritis undergoing primary hip or knee arthroplasty: prospective, two-arm, parallel trial. J Med Internet Res. 2021;23:e28320. 10.2196/28320. 34473068 PMC8446839

[2] Zhao C, Liao Q, Yang D, Yang M, Xu P. Advances in perioperative pain management for total knee arthroplasty: a review of multimodal analgesic approaches. J Orthop Surg Res. 2024;19:843. 10.1186/s13018-024-05324-4. 39696522 PMC11658298

[3] Awad G, Saad J-P, Hamyeh A, Boutros M. Efficacy and safety of intra-articular mesenchymal stem cell–based therapies in knee osteoarthritis: a systematic review and meta-analysis of randomized controlled trials. Clin Rheumatol. 2026;45:2905–42. 10.1007/s10067-026-08042-w. 41863718

[4] Dawson Z, Stanton SS, Roy S, Farjo R, Aslesen HA, Hallstrom BR, et al. Opioid consumption after discharge from total knee and hip arthroplasty: a systematic review and meta-analysis. J Arthroplasty. 2024;39:2130-2136.e7. 10.1016/j.arth.2024.01.063. 38336301

[5] Ramtin S. Oral multi-modal agents for post-operative pain management: a current concept. SurgiColl. 2025. 10.58616/001c.130902.

[6] Berardino K, Carroll AH, Ricotti R, Popovsky D, Civilette MD, Urits I, et al. The ramifications of opioid utilization and outcomes of alternative pain control strategies for total knee arthroplasties. Orthop Rev. 2022. 10.52965/001c.37496. PMC9425522 36045694

[7] Yuan M, Tang T, Ding Z, Li H, Zhou Z. Analgesic effect of perioperative duloxetine in patients after total knee arthroplasty: a prospective, randomized, double-blind, placebo-controlled trial. BMC Musculoskelet Disord. 2022;23:242. 10.1186/s12891-022-05194-z. 35279155 PMC8917721

[8] Jones IA, Talehakimi A, Murphy LS, Wang JC, Piple AS, Christ AB, et al. Duloxetine for postoperative pain control following knee or hip replacement: a systematic review and meta-analysis. Arthroplast Today. 2023;20:101097. 10.1016/j.artd.2023.101097. 36852213 PMC9957748

[9] Lin Y, Jiang M, Liao C, Wu Q, Zhao J. Duloxetine reduces opioid consumption and pain after total hip or knee arthroplasty: a meta-analysis of randomized controlled trials. J Orthop Surg Res. 2024;19:181. 10.1186/s13018-024-04648-5. 38481321 PMC10936099

[10] Imani F, Emami A, Alimian M, Nikoubakht N, Khosravi N, Rajabi M, et al. Comparison of perioperative pregabalin and duloxetine for pain management after total knee arthroplasty: a double-blind clinical trial. Anesthesiol Pain Med. 2023. 10.5812/aapm-127017. PMC10389034 37529346

[11] Rienstra W, Blikman T, Dijkstra B, Stewart R, Zijlstra W, van Raaij T, et al. Effect of preoperative duloxetine treatment on postoperative chronic residual pain after total hip or knee arthroplasty: a randomised controlled trial. BMJ Open. 2021;11:e052944. 10.1136/bmjopen-2021-052944. 34732491 PMC8572398

[12] Ding Z, Li H, Huang C, Yuan M, Cao J, Wang H, et al. Significant analgesic benefits of perioperative duloxetine in patients who have depressive symptoms undergoing total hip arthroplasty: a randomized controlled trial. J Arthroplasty. 2023;38:519–24. 10.1016/j.arth.2022.10.007. 36252745

[13] Koh IJ, Kim MS, Sohn S, Song KY, Choi NY, In Y. Duloxetine reduces pain and improves quality of recovery following total knee arthroplasty in centrally sensitized patients. J Bone Jt Surg. 2019;101:64–73. 10.2106/JBJS.18.00347. 30601417

[14] Kim MS, Koh IJ, Sung YG, Park DC, Na JW, In Y. Preemptive duloxetine relieves postoperative pain and lowers wound temperature in centrally sensitized patients undergoing total knee arthroplasty: a randomized, double-blind, placebo-controlled trial. J Clin Med. 2021;10:2809. 10.3390/jcm10132809. 34202314 PMC8269433

[15] Li H, Zeng W-N, Ding Z-C, Yuan M-C, Cai Y-R, Zhou Z-K. Duloxetine reduces pain after total hip arthroplasty: a prospective, randomized controlled study. BMC Musculoskelet Disord. 2021;22:492. 10.1186/s12891-021-04377-4. 34049519 PMC8161627

[16] Inamullah AH, Essa MA, Farooq MZ, Ullah S, Gul Y. Effect of duloxetine on pain relief in patients undergoing arthroplasty. Med Forum Mon. 2023;34:63–7.

[17] Pinsornsak P, Phunphakchit J, Pinsornsak P, Boontanapibul K. Does postoperative low-dose duloxetine provide analgesic effect and lower morphine consumption after primary total knee arthroplasty? A prospective, double-blind, randomized controlled trial. Arch Orthop Trauma Surg. 2024;144:4979–87. 10.1007/s00402-024-05591-0. 39347965

[18] Rajani AM, Mittal ARS, Kulkarni VU, Desai MK, Dubey RR, Rajani KA. Duloxetine as an analgesic in patients who do not have central sensitivity undergoing single-setting, bilateral total knee arthroplasty: a prospective, double-blinded, randomized, placebo-controlled trial. J Arthroplasty. 2024;39:2055–60. 10.1016/j.arth.2024.02.007. 38355065

[19] Ho K-Y, Tay W, Yeo M-C, Liu H, Yeo S-J, Chia S-L, et al. Duloxetine reduces morphine requirements after knee replacement surgery. Br J Anaesth. 2010;105:371–6. 10.1093/bja/aeq158. 20573635

[20] YaDeau JT, Brummett CM, Mayman DJ, Lin Y, Goytizolo EA, Padgett DE, et al. Duloxetine and subacute pain after knee arthroplasty when added to a multimodal analgesic regimen. Anesthesiology. 2016;125:561–72. 10.1097/ALN.0000000000001228. 27387351

[21] YaDeau JT, Mayman DJ, Jules-Elysee KM, Lin Y, Padgett DE, DeMeo DA, et al. Effect of duloxetine on opioid use and pain after total knee arthroplasty: a triple-blinded randomized controlled trial. J Arthroplasty. 2022;37:S147–54. 10.1016/j.arth.2022.02.022. 35346549

[22] Miaskowski C, Bair M, Chou R, D’Arcy Y, Hartrick C, Huffman L, et al. Principles of analgesic use in the treatment of acute pain and cancer pain. 6th ed. Glenview, IL: American Pain Society; 2008.

[23] Ramadanov N, Voss M, Diallo RM, Lettner J, Hakam HT, Prill R, et al. Do meta‐analyses of total hip arthroplasty produce reliable results? A systematic review and meta‐epidemiological study of statistical methods. Orthop Surg. 2025;17:1936–55. 10.1111/os.70077. 40425483 PMC12214412

[24] Laigaard J, Karlsen A, Maagaard M, Haxholdt Lunn T, Mathiesen O, Overgaard S. Perioperative analgesic interventions for reduction of persistent postsurgical pain after total hip and knee arthroplasty: a systematic review and meta-analysis. Anesth Analg. 2025;141:765–78. 10.1213/ANE.0000000000007246. 39418206

[25] Boutros M, Awad G, Mjahed GA, Mansour E. Simultaneous bilateral total knee arthroplasty lowers reoperation and cost at the expense of higher complications and mortality: a meta-analysis and systematic review. Knee Surg Relat Res. 2025;37:45. 10.1186/s43019-025-00297-y. 41126378 PMC12548196

[26] Kamdar MM. Principles of analgesic use in the treatment of acute pain and cancer pain, sixth edition. J Palliat Med. 2010;13:217–8. 10.1089/jpm.2010.9854.

[27] Branton MW, Hopkins TJ, Nemec EC. Duloxetine for the reduction of opioid use in elective orthopedic surgery: a systematic review and meta-analysis. Int J Clin Pharm. 2021;43:394–403. 10.1007/s11096-020-01216-9. 33459948

[28] Azimi A, Hooshmand E, Mafi AA, Tabatabaei F-S. Effect of duloxetine on opioid consumption and pain after total knee and hip arthroplasty: a systematic review and meta-analysis of randomized clinical trials. Pain Med. 2023;24:1035–45. 10.1093/pm/pnad045. 37027215

[29] Zhong H, Li J, Chen Y, Huang Y, Wen Z, Zhao J. Effect of duloxetine on pain and opioid consumption after total knee and hip arthroplasty: a systematic review and meta-analysis of randomized controlled trials. Int J Clin Pharm. 2024;46:14–25. 10.1007/s11096-023-01593-x. 37294475

[30] Zhou Y, Chen X, Chen C, Cao Y. The efficacy and safety of duloxetine for the treatment of patients after TKA or THA: a systematic review and meta-analysis. Medicine. 2023;102:e34895. 10.1097/MD.0000000000034895. 37653762 PMC10470761

[31] Yang J-M, Wang Y, Li J-Y, Li C-C, Wang Z-T, Shen Z, et al. Duloxetine for rehabilitation after total knee arthroplasty: a systematic review and meta-analysis. Int J Surg. 2023;109:913–24. 10.1097/JS9.0000000000000230. 37097617 PMC10389646

[32] Zhang L-K, Li Q, Fang Y-F, Qi J-W. Effect of duloxetine on pain relief after total knee arthroplasty: a meta-analysis of randomized controlled trials. Medicine. 2023;102:e33101. 10.1097/MD.0000000000033101. 36897714 PMC9997837

[33] Boutros M, Awad G, Abboud E, Zhao A, Thakkar SC. Total knee arthroplasty with or without a tourniquet: a meta-analysis of randomized controlled trials. Eur J Orthop Surg Traumatol. 2025;35:397. 10.1007/s00590-025-04492-1. 40946110

[34] Alasbali MS, Alshammari YA, Alhomaidi MS, Alaqil IA, Sharif MA, Al-Shahir AM, et al. Preoperative central sensitization as a predictor of pain outcomes after hip and knee arthroplasty: a systematic review. Cureus. 2026. 10.7759/cureus.101791. 41710828 PMC12910524

[35] Chan B, Ward S, Abdallah FW, Jones C, Papachristos A, Chin K, et al. Opioid prescribing and utilization patterns in patients having elective hip and knee arthroplasty: association between prescription patterns and opioid consumption. Can J Anaesth. 2022;69:953–62. 10.1007/s12630-021-02145-5. 34873641

[36] Boutros M, Awad G, Mouawad A, Mansour E. Kinematic versus mechanically aligned total knee arthroplasty: a meta-analysis of randomized controlled trials. J Knee Surg. 2026;39:283–95. 10.1055/a-2741-1246. 41285388

[37] Boutros M, Awad G, Hassan C, Hammad S, Assi J, Haykal G, et al. Cross-cultural adaptation and psychometric validation of the first Arabic KOOS-12: a reliable tool for assessing knee outcomes in Arabic-speaking populations. Knee Surg Relat Res. 2026;38:16. 10.1186/s43019-026-00316-6. 41964099 PMC13067642

