# Supplementary material for: Duloxetine for postoperative pain management in total hip and knee arthroplasty: a systematic review and meta-analysis
Source: J Orthop Surg Res. 2026 Sep 2;21:527. doi: 10.1186/s13018-026-07176-6 (PMC13536615; doi:10.1186/s13018-026-07176-6)
Supplement: Supplementary file 1 [file 13018_2026_7176_MOESM1_ESM.docx]

| **Section and Topic** | **Item #** | **Checklist item** | **Location where item is reported** |
| --- | --- | --- | --- |
| **TITLE** | | |  |
| Title | 1 | Identify the report as a systematic review. | Title |
| **ABSTRACT** | | |  |
| Abstract | 2 | See the PRISMA 2020 for Abstracts checklist. | Abstract |
| **INTRODUCTION** | | |  |
| Rationale | 3 | Describe the rationale for the review in the context of existing knowledge. | Introduction, Paragraphs 1–4 |
| Objectives | 4 | Provide an explicit statement of the objective(s) or question(s) the review addresses. | Introduction, Paragraph 5 |
| **METHODS** | | |  |
| Eligibility criteria | 5 | Specify the inclusion and exclusion criteria for the review and how studies were grouped for the syntheses. | Methods, Eligibility Criteria section, Paragraph 1 |
| Information sources | 6 | Specify all databases, registers, websites, organisations, reference lists and other sources searched or consulted to identify studies. Specify the date when each source was last searched or consulted. | Methods, Paragraph 1 (Materials and Methods opening paragraph) |
| Search strategy | 7 | Present the full search strategies for all databases, registers and websites, including any filters and limits used. | Methods, Paragraph 1 (Materials and Methods opening paragraph); full strategies in Additional File 2: Table S1 |
| Selection process | 8 | Specify the methods used to decide whether a study met the inclusion criteria of the review, including how many reviewers screened each record and each report retrieved, whether they worked independently, and if applicable, details of automation tools used in the process. | Methods, Screening and Data Extraction section, Paragraph 1 |
| Data collection process | 9 | Specify the methods used to collect data from reports, including how many reviewers collected data from each report, whether they worked independently, any processes for obtaining or confirming data from study investigators, and if applicable, details of automation tools used in the process. | Methods, Screening and Data Extraction section, Paragraph 1 |
| Data items | 10a | List and define all outcomes for which data were sought. Specify whether all results that were compatible with each outcome domain in each study were sought (e.g. for all measures, time points, analyses), and if not, the methods used to decide which results to collect. | Methods, Eligibility Criteria section, Paragraph 1 |
|  | 10b | List and define all other variables for which data were sought (e.g. participant and intervention characteristics, funding sources). Describe any assumptions made about any missing or unclear information. | Methods, Screening and Data Extraction section, Paragraph 1 |
| Study risk of bias assessment | 11 | Specify the methods used to assess risk of bias in the included studies, including details of the tool(s) used, how many reviewers assessed each study and whether they worked independently, and if applicable, details of automation tools used in the process. | Methods, Screening and Data Extraction section, Paragraph 1 |
| Effect measures | 12 | Specify for each outcome the effect measure(s) (e.g. risk ratio, mean difference) used in the synthesis or presentation of results. | Methods, Statistical Analysis section, Paragraphs 1–2 |
| Synthesis methods | 13a | Describe the processes used to decide which studies were eligible for each synthesis (e.g. tabulating the study intervention characteristics and comparing against the planned groups for each synthesis (item #5)). | Methods, Eligibility Criteria section, Paragraph 1; Methods, Statistical Analysis section, Paragraphs 1–2 |
|  | 13b | Describe any methods required to prepare the data for presentation or synthesis, such as handling of missing summary statistics, or data conversions. | Methods, Statistical Analysis section, Paragraphs 1–2 |
|  | 13c | Describe any methods used to tabulate or visually display results of individual studies and syntheses. | Methods, Statistical Analysis section, Paragraph 1; Results, Table 1 and Table 2 |
|  | 13d | Describe any methods used to synthesize results and provide a rationale for the choice(s). If meta-analysis was performed, describe the model(s), method(s) to identify the presence and extent of statistical heterogeneity, and software package(s) used. | Methods, Statistical Analysis section, Paragraphs 1–2 |
|  | 13e | Describe any methods used to explore possible causes of heterogeneity among study results (e.g. subgroup analysis, meta-regression). | Methods, Statistical Analysis section, Paragraph 1 |
|  | 13f | Describe any sensitivity analyses conducted to assess robustness of the synthesized results. | Methods, Statistical Analysis section, Paragraph 2 |
| Reporting bias assessment | 14 | Describe any methods used to assess risk of bias due to missing results in a synthesis (arising from reporting biases). | Methods, Statistical Analysis section, Paragraph 2 |
| Certainty assessment | 15 | Describe any methods used to assess certainty (or confidence) in the body of evidence for an outcome. | Not explicitly reported as a formal certainty assessment; risk of bias is addressed via RoB 2 tool in Screening and Data Extraction section |
| **RESULTS** | | |  |
| Study selection | 16a | Describe the results of the search and selection process, from the number of records identified in the search to the number of studies included in the review, ideally using a flow diagram. | Results, Search Results and Characteristics of Included Studies section, Paragraph 1; Figure 1 |
|  | 16b | Cite studies that might appear to meet the inclusion criteria, but which were excluded, and explain why they were excluded. | Results, Search Results and Characteristics of Included Studies section, Paragraph 1 |
| Study characteristics | 17 | Cite each included study and present its characteristics. | Results, Search Results and Characteristics of Included Studies section, Paragraph 1; Table 1; Table 2 |
| Risk of bias in studies | 18 | Present assessments of risk of bias for each included study. | Results, Quality Assessment section, Paragraphs 1–4; Figures 2 and 3 |
| Results of individual studies | 19 | For all outcomes, present, for each study: (a) summary statistics for each group (where appropriate) and (b) an effect estimate and its precision (e.g. confidence/credible interval), ideally using structured tables or plots. | Results, Primary Outcomes and Secondary Outcomes sections; Figures 4–7 |
| Results of syntheses | 20a | For each synthesis, briefly summarise the characteristics and risk of bias among contributing studies. | Results, Primary Outcomes and Secondary Outcomes sections; Discussion, Summary of Main Findings section |
|  | 20b | Present results of all statistical syntheses conducted. If meta-analysis was done, present for each the summary estimate and its precision (e.g. confidence/credible interval) and measures of statistical heterogeneity. If comparing groups, describe the direction of the effect. | Results, Primary Outcomes section; Secondary Outcomes section; Figures 4–7 |
|  | 20c | Present results of all investigations of possible causes of heterogeneity among study results. | Results, Primary Outcomes section (≥3-month Temporal Subgroup Analysis); Figure 5; Discussion, Limitations section — Heterogeneity subsection |
|  | 20d | Present results of all sensitivity analyses conducted to assess the robustness of the synthesized results. | Results, Sensitivity Analysis section, Paragraph 1; Additional File 3: Tables S2–4 |
| Reporting biases | 21 | Present assessments of risk of bias due to missing results (arising from reporting biases) for each synthesis assessed. | Results, Publication Bias section, Paragraphs 1–4; Figures 8 and 9 |
| Certainty of evidence | 22 | Present assessments of certainty (or confidence) in the body of evidence for each outcome assessed. | Discussion, Limitations section — Risk of Bias in Included Studies subsection |
| **DISCUSSION** | | |  |
| Discussion | 23a | Provide a general interpretation of the results in the context of other evidence. | Discussion, Summary of Main Findings; Agreement with Existing Literature; Nuanced Findings and Points of Divergence; Novel Contributions of the Present Meta-Analysis sections |
|  | 23b | Discuss any limitations of the evidence included in the review. | Discussion, Limitations section — Risk of Bias in Included Studies; Variability in Background Analgesic Regimens (Additional file 4: Table S5); Heterogeneity of Patient Populations subsections |
|  | 23c | Discuss any limitations of the review processes used. | Discussion, Limitations section — Heterogeneity and Inability to Perform Subgroup Analyses subsections |
|  | 23d | Discuss implications of the results for practice, policy, and future research. | Discussion, Clinical Implications section; Future Research Directions section |
| **OTHER INFORMATION** | | |  |
| Registration and protocol | 24a | Provide registration information for the review, including register name and registration number, or state that the review was not registered. | Abstract, Methods paragraph; Methods, opening paragraph |
|  | 24b | Indicate where the review protocol can be accessed, or state that a protocol was not prepared. | Methods, opening paragraph |
|  | 24c | Describe and explain any amendments to information provided at registration or in the protocol. | Not reported |
| Support | 25 | Describe sources of financial or non-financial support for the review, and the role of the funders or sponsors in the review. | Declarations, Funding section |
| Competing interests | 26 | Declare any competing interests of review authors. | Declarations, Competing Interests section |
| Availability of data, code and other materials | 27 | Report which of the following are publicly available and where they can be found: template data collection forms; data extracted from included studies; data used for all analyses; analytic code; any other materials used in the review. | Declarations, Availability of Data and Materials section |
