# Supplementary material for: Duloxetine for postoperative pain management in total hip and knee arthroplasty: a systematic review and meta-analysis
Source: J Orthop Surg Res. 2026 Sep 2;21:527. doi: 10.1186/s13018-026-07176-6 (PMC13536615; doi:10.1186/s13018-026-07176-6)
Supplement: Supplementary file 2 [file 13018_2026_7176_MOESM2_ESM.docx]

**Table S1. Search strategy**

Full Boolean search strategies used across PubMed, Web of Science, Scopus, and the Cochrane Library, including search terms, operators, date filters, and total results retrieved per database (searched from inception to March 2, 2026).

| Database | Strategy | Results |
| --- | --- | --- |
| PubMed | #1 Dulox* OR Dulot OR DXT OR Cymbalta OR Yentreve OR Drizalma OR Irenka OR Xeristar OR Symbal  #2 Arthroplast* OR Replacement OR Prosthesis  #3 #1 AND #2  #4 #3 AND ("1900/01/01"[Date - Publication] : "2026/04/01"[Date - Publication]) | 106 |
| Web of Science | #1 TS=(Dulox* OR Dulot OR DXT OR Cymbalta OR Yentreve OR Drizalma OR Irenka OR Xeristar OR Symbal)  #2 TS=(Arthroplast* OR Replacement OR Prosthesis)  #3 #1 AND #2  #4 #3 AND DOP=(1900-01-01/2026-04-01) | 123 |
| Scopus | #1 TITLE-ABS-KEY(Dulox* OR Dulot OR DXT OR Cymbalta OR Yentreve OR Drizalma OR Irenka OR Xeristar OR Symbal)  #2 TITLE-ABS-KEY(Arthroplast* OR Replacement OR Prosthesis)  #3 #1 AND #2  #4 #3 AND PUBYEAR > 1899 AND PUBYEAR < 2027 | 350 |
| Cochrane Library | #1 Dulox* OR Dulot OR DXT OR Cymbalta OR Yentreve OR Drizalma OR Irenka OR Xeristar OR Symbal  #2 Arthroplast* OR Replacement OR Prosthesis  #3 #1 AND #2  #4 #3 with Cochrane Library publication date Between Jan 1900 and Apr 2026 | 36 |

---

Explanation of full search strategy for PubMed (example)

(("duloxetine"[MeSH Terms] OR "duloxetine"[All Fields] OR "duloxetines"[All Fields] OR "dulox"[All Fields] OR "dulot"[All Fields] OR "dxt"[All Fields] OR "cymbalta"[All Fields] OR "yentreve"[All Fields] OR "drizalma"[All Fields] OR "irenka"[All Fields] OR "xeristar"[All Fields] OR "symbal"[All Fields])

AND

("arthroplasty"[MeSH Terms] OR "arthroplasty"[All Fields] OR "arthroplasties"[All Fields] OR "arthroplastic"[All Fields] OR "arthroplastics"[All Fields] OR "replace"[All Fields] OR "replaceable"[All Fields] OR "replaced"[All Fields] OR "replaces"[All Fields] OR "replacing"[All Fields] OR "replacement"[All Fields] OR "replantation"[MeSH Terms] OR "replantation"[All Fields] OR "replacements"[All Fields] OR "prosthesis"[MeSH Terms] OR "prosthesis"[All Fields] OR "prostheses"[All Fields] OR "prosthetic"[All Fields] OR "prosthetics"[All Fields] OR "prosthetist"[All Fields] OR "prosthetists"[All Fields]))

AND 1900/01/01:2026/04/01[Date - Publication]
