# Supplementary material for: Duloxetine for postoperative pain management in total hip and knee arthroplasty: a systematic review and meta-analysis
Source: J Orthop Surg Res. 2026 Sep 2;21:527. doi: 10.1186/s13018-026-07176-6 (PMC13536615; doi:10.1186/s13018-026-07176-6)
Supplement: Supplementary file 3 [file 13018_2026_7176_MOESM3_ESM.docx]

**Table (S2) : Leave-One-Out Sensitivity Analysis for Postoperative Pain Scores**

| **Outcome / Omitted Study** | **MD** | **CI min** | **CI max** |
| --- | --- | --- | --- |
| **Resting pain score 24h** |  |  |  |
| Original | -5.09 | -8.76 | -1.43 |
| Omitted Ho K-Y 2010 | -5.3 | -9.11 | -1.48 |
| Omitted YaDeau JT 2016 | -5.64 | -10.41 | -0.86 |
| Omitted Koh IJ 2019 | -5.12 | -9.03 | -1.21 |
| Omitted Kim MS 2021 | -3.66 | -6.81 | -0.5 |
| Omitted Li H 2021 | -5.16 | -9.4 | -0.93 |
| Omitted YaDeau JT 2022 | -6.24 | -9.97 | -2.52 |
| Omitted Yuan M 2022 | -4.56 | -8.33 | -0.79 |
| Omitted Ding 2023 | -5.89 | -9.86 | -1.92 |
| Omitted Imani 2023 | -3.99 | -7.47 | -0.5 |
| Omitted Inamullah 2023 ‡ | -5.28 | -9.48 | -1.08 |
| Omitted Pinsornsak 2024 | -5.31 | -9.26 | -1.35 |
| **Outcome / Omitted Study** | **MD** | **CI min** | **CI max** |
| **Ampulating pain score 24h** |  |  |  |
| Original | -4.55 | -8.71 | -0.4 |
| Omitted Ho K-Y 2010 | -4.96 | -9.64 | -0.28 |
| Omitted YaDeau JT 2016 | -4.26 | -8.5 | -0.02 |
| Omitted Koh IJ 2019 | -2.44 | -5.19 | 0.3 |
| Omitted Kim MS 2021 | -4.56 | -9.21 | 0.09 |
| Omitted Li H 2021 | -4.64 | -9.71 | 0.44 |
| Omitted YaDeau JT 2022 | -5.71 | -10.01 | -1.41 |
| Omitted Yuan M 2022 | -5.44 | -10.79 | -0.09 |
| Omitted Ding 2023 | -5.29 | -9.89 | -0.7 |
| Omitted Pinsornsak 2024 | -4.04 | -8.45 | 0.38 |
| **Outcome / Omitted Study** | **MD** | **CI min** | **CI max** |
| **Resting pain score 2w** |  |  |  |
| Original | -6.48 | -11.14 | -1.82 |
| Omitted YaDeau JT 2016 | -7.74 | -11.53 | -3.95 |
| Omitted Koh IJ 2019 | -5.48 | -10.09 | -0.86 |
| Omitted Kim MS 2021 | -5.4 | -10.04 | -0.76 |
| Omitted YaDeau JT 2022 | -6.75 | -12 | -1.49 |
| Omitted Ding 2023 | -6.43 | -11.74 | -1.12 |
| Omitted Pinsornsak 2024 | -7.6 | -13 | -2.2 |
| Omitted Rajani 2024 † | -5.97 | -10.98 | -0.97 |
| **Outcome / Omitted Study** | **MD** | **CI min** | **CI max** |
| **Ampulating pain score 2w** |  |  |  |
| Original | -8.93 | -13.16 | -4.69 |
| Omitted YaDeau JT 2016 | -10.05 | -14.56 | -5.53 |
| Omitted Koh IJ 2019 | -7.19 | -10.68 | -3.71 |
| Omitted Kim MS 2021 | -8.49 | -12.95 | -4.03 |
| Omitted YaDeau JT 2022 | -9.42 | -14.23 | -4.61 |
| Omitted Ding 2023 | -8.25 | -12.72 | -3.78 |
| Omitted Pinsornsak 2024 | -10.1 | -14.93 | -5.27 |
| Omitted Rajani 2024 † | -9.01 | -14.24 | -3.78 |
| **Outcome / Omitted Study** | **MD** | **CI min** | **CI max** |
| **Resting pain score ≥ 3m** |  |  |  |
| Original | -2.79 | -4.93 | -0.64 |
| Omitted Koh IJ 2019 | -2.1 | -4.09 | -0.12 |
| Omitted Kim MS 2021 | -2.59 | -4.81 | -0.37 |
| Omitted Li H 2021 | -3.3 | -5.92 | -0.68 |
| Omitted Rienstra W 2021 | -2.83 | -5.01 | -0.65 |
| Omitted Yuan M 2022 | -3.2 | -5.95 | -0.44 |
| Omitted Ding 2023 | -3.45 | -5.74 | -1.17 |
| Omitted Inamullah 2023 ‡ | -1.63 | -3.28 | 0.02 |
| Omitted Pinsornsak 2024 | -3.04 | -5.54 | -0.54 |
| Omitted Rajani 2024 † | -3.27 | -5.72 | -0.81 |
| **Outcome / Omitted Study** | **MD** | **CI min** | **CI max** |
| **Ampulating pain score ≥ 3m** |  |  |  |
| Original | -3.76 | -6.62 | -0.89 |
| Omitted Koh IJ 2019 | -2.26 | -3.9 | -0.61 |
| Omitted Kim MS 2021 | -3.3 | -6.29 | -0.3 |
| Omitted Li H 2021 | -4.16 | -7.55 | -0.78 |
| Omitted Rienstra W 2021 | -3.81 | -6.74 | -0.88 |
| Omitted Yuan M 2022 | -4.13 | -7.96 | -0.3 |
| Omitted Ding 2023 | -3.8 | -7.16 | -0.45 |
| Omitted Pinsornsak 2024 | -4.48 | -7.57 | -1.39 |
| Omitted Rajani 2024 † | -4.52 | -7.85 | -1.19 |

**Table (S3) : Leave-One-Out Sensitivity Analysis for Postoperative Opioid Consumption**

| **Outcome / Omitted Study** | **MD** | **CI min** | **CI max** |
| --- | --- | --- | --- |
| **Opioid consumption 24h** |  |  |  |
| Original | -6.24 | -15.39 | 2.92 |
| Omitted Ho K-Y 2010 | -4.72 | -14.36 | 4.92 |
| Omitted YaDeau JT 2016 | -3.51 | -10.77 | 3.74 |
| Omitted Li H 2021 | -5.60 | -16.00 | 4.80 |
| Omitted YaDeau JT 2022 | -8.88 | -15.59 | -2.17 |
| Omitted Yuan M 2022 | -6.84 | -18.06 | 4.38 |
| Omitted Pinsornsak 2024 | -7.15 | -18.52 | 4.22 |
| **Outcome / Omitted Study** | **MD** | **CI min** | **CI max** |
| **Opioid consumption 2d** |  |  |  |
| Original | -13.88 | -33.76 | 5.99 |
| Omitted Ho K-Y 2010 | -22.74 | -44.17 | -1.31 |
| Omitted YaDeau JT 2016 | -28.45 | -34.95 | -21.95 |
| Omitted YaDeau JT 2022 | -9.01 | -33.21 | 15.19 |
| Omitted Yuan M 2022 | -8.97 | -33.16 | 15.22 |
| Omitted Pinsornsak 2024 | -8.39 | -31.29 | 14.52 |
| **Outcome / Omitted Study** | **MD** | **CI min** | **CI max** |
| **Opioid consumption 3d** |  |  |  |
| Original | -10.62 | -17.89 | -3.34 |
| Omitted YaDeau JT 2016 | -8.81 | -16.26 | -1.36 |
| Omitted Li H 2021 | -10.58 | -18.67 | -2.49 |
| Omitted YaDeau JT 2022 | -8.64 | -17.26 | -0.01 |
| Omitted Yuan M 2022 | -12.39 | -20.37 | -4.40 |
| Omitted Pinsornsak 2024 | -12.65 | -20.45 | -4.84 |
| **Outcome / Omitted Study** | **MD** | **CI min** | **CI max** |
| **Opioid consumption 7d** |  |  |  |
| Original | -9.44 | -20.61 | 1.73 |
| Omitted Li H 2021 | -8.00 | -21.00 | 5.01 |
| Omitted YaDeau JT 2022 | -5.81 | -17.44 | 5.82 |
| Omitted Yuan M 2022 | -14.65 | -16.83 | -12.46 |

**Table (S4) : Leave-One-Out Sensitivity Analysis for Adverse Effects**

| **Outcome / Omitted Study** | **RR** | **CI min** | **CI max** |
| --- | --- | --- | --- |
| **Nausea/Vomiting** |  |  |  |
| Original | 0.73 | 0.55 | 0.97 |
| Omitted Ho K-Y 2010 | 0.73 | 0.55 | 0.99 |
| Omitted YaDeau JT 2016 | 0.72 | 0.54 | 0.96 |
| Omitted Koh IJ 2019 | 0.66 | 0.48 | 0.91 |
| Omitted Kim MS 2021 | 0.74 | 0.55 | 1 |
| Omitted Li H 2021 | 0.69 | 0.51 | 0.94 |
| Omitted Yuan M 2022 | 0.79 | 0.58 | 1.07 |
| Omitted YaDeau JT 2022 | 0.72 | 0.53 | 0.98 |
| Omitted Ding 2023 | 0.78 | 0.57 | 1.06 |
| Omitted Inamullah 2023 ‡ | 0.75 | 0.55 | 1.02 |
| Omitted Pinsornsak 2024 | 0.7 | 0.52 | 0.94 |
| Omitted Rajani 2024 † | 0.74 | 0.55 | 1 |
| **Outcome / Omitted Study** | **RR** | **CI min** | **CI max** |
| **Fatigue** |  |  |  |
| Original | 0.87 | 0.76 | 1 |
| Omitted Koh IJ 2019 | 0.8 | 0.64 | 0.99 |
| Omitted Kim MS 2021 | 0.86 | 0.75 | 0.98 |
| Omitted Li H 2021 | 0.87 | 0.76 | 1 |
| Omitted Yuan M 2022 | 0.87 | 0.75 | 1 |
| Omitted Ding 2023 | 0.86 | 0.75 | 0.99 |
| Omitted Inamullah 2023 ‡ | 0.91 | 0.78 | 1.06 |
| Omitted Rajani 2024 † | 0.88 | 0.76 | 1.01 |
| **Outcome / Omitted Study** | **RR** | **CI min** | **CI max** |
| **Dry mouth** |  |  |  |
| Original | 0.92 | 0.73 | 1.17 |
| Omitted Koh IJ 2019 | 0.9 | 0.69 | 1.18 |
| Omitted Kim MS 2021 | 0.97 | 0.76 | 1.24 |
| Omitted Li H 2021 | 0.91 | 0.72 | 1.16 |
| Omitted Yuan M 2022 | 0.89 | 0.69 | 1.17 |
| Omitted Ding 2023 | 0.92 | 0.72 | 1.16 |
| Omitted Inamullah 2023 ‡ | 0.95 | 0.72 | 1.26 |
| Omitted Pinsornsak 2024 | 0.93 | 0.72 | 1.19 |
| Omitted Rajani 2024 † | 0.93 | 0.73 | 1.18 |
| **Outcome / Omitted Study** | **RR** | **CI min** | **CI max** |
| **Constipation** |  |  |  |
| Original | 0.86 | 0.65 | 1.14 |
| Omitted Koh IJ 2019 | 0.76 | 0.54 | 1.08 |
| Omitted Kim MS 2021 | 0.86 | 0.62 | 1.18 |
| Omitted Li H 2021 | 0.87 | 0.65 | 1.16 |
| Omitted Yuan M 2022 | 0.92 | 0.69 | 1.22 |
| Omitted Ding 2023 | 0.84 | 0.63 | 1.11 |
| Omitted Inamullah 2023 ‡ | 0.88 | 0.66 | 1.17 |
| Omitted Pinsornsak 2024 | 0.86 | 0.65 | 1.15 |
| Omitted Rajani 2024 † | 0.87 | 0.65 | 1.16 |
| **Outcome / Omitted Study** | **RR** | **CI min** | **CI max** |
| **Dizziness** |  |  |  |
| Original | 0.94 | 0.61 | 1.46 |
| Omitted Ho K-Y 2010 | 0.96 | 0.61 | 1.51 |
| Omitted Koh IJ 2019 | 0.88 | 0.57 | 1.38 |
| Omitted Kim MS 2021 | 0.88 | 0.54 | 1.43 |
| Omitted Li H 2021 | 0.96 | 0.61 | 1.52 |
| Omitted Yuan M 2022 | 0.99 | 0.62 | 1.57 |
| Omitted Ding 2023 | 0.94 | 0.59 | 1.49 |
| Omitted Inamullah 2023 ‡ | 1.11 | 0.69 | 1.81 |
| Omitted Pinsornsak 2024 | 0.82 | 0.51 | 1.3 |
| Omitted Rajani 2024 † | 0.96 | 0.61 | 1.52 |
| **Outcome / Omitted Study** | **RR** | **CI min** | **CI max** |
| **Drowsiness/Somnolence** |  |  |  |
| Original | 1.88 | 1.28 | 2.78 |
| Omitted Ho K-Y 2010 | 1.97 | 1.33 | 2.92 |
| Omitted Li H 2021 | 1.87 | 1.25 | 2.81 |
| Omitted YaDeau JT 2022 | 2.01 | 1.26 | 3.22 |
| Omitted Yuan M 2022 | 1.82 | 1.13 | 2.95 |
| Omitted Ding 2023 | 1.88 | 1.25 | 2.82 |
| Omitted Rajani 2024 † | 1.76 | 1.16 | 2.66 |

†Rajani et al. (2024) enrolled patients undergoing single-setting bilateral TKA and was subjected to a dedicated sensitivity analysis in addition to the standard leave-one-out procedure. This study contributed data to rVAS and aVAS analyses at 2 weeks and ≥3 months, and to adverse effect outcomes; it did not contribute to 24-hour VAS or any opioid consumption analyses, and consequently does not appear as an omitted-study row in those sections of this table.

‡Inamullah et al. (2023) was subjected to a dedicated sensitivity analysis in view of methodological concerns (open-label design, blinding not reported). This study contributed data to rVAS at 24 hours, rVAS at ≥3 months, and to the following adverse effect outcomes: nausea/vomiting, fatigue, dry mouth, constipation, and dizziness. It did not contribute data to aVAS at any timepoint, to opioid consumption outcomes at any timepoint, or to drowsiness/somnolence, and consequently does not appear as an omitted-study row in those sections of this table.
