# Supplementary material for: Duloxetine for postoperative pain management in total hip and knee arthroplasty: a systematic review and meta-analysis
Source: J Orthop Surg Res. 2026 Sep 2;21:527. doi: 10.1186/s13018-026-07176-6 (PMC13536615; doi:10.1186/s13018-026-07176-6)
Supplement: Supplementary file 4 [file 13018_2026_7176_MOESM4_ESM.docx]

**Table (S5) : Background analgesic regimens and rescue analgesia protocols for each included trial**

| **Study** | **Anesthesia type** | **Regional block / LIA** | **Scheduled peri-operative analgesics** | **Intra-operative adjuncts** | **Rescue analgesia** |
| --- | --- | --- | --- | --- | --- |
| Ho K-Y 2010 | Spinal or GA | None | PCM 1 g q6h; NSAIDs prohibited | IV morphine ≤0.1 mg/kg intraop | PCA IV morphine (1 mg bolus, 5-min lockout) |
| YaDeau JT 2016 | CSE | ACB (bupivacaine + dexamethasone) | Meloxicam 15 mg/d; PCEA (bupivacaine/hydromorphone) until POD1 | IV ketorolac; IV dexamethasone 4 mg | PO oxycodone/PCM PRN |
| Koh IJ 2019 | GA | None | Celecoxib; pregabalin; oxycodone; tramadol; PCM (post-op scheduled) | Tourniquet 300 mmHg | PCA IV fentanyl (on-demand only) |
| Kim MS 2021 | GA | LIA (ropivacaine/morphine/ketorolac) | Celecoxib; pregabalin; oxycodone; tramadol; PCM (post-op scheduled) | Tourniquet 300 mmHg | PCA IV fentanyl; IM tramadol (VAS >6) |
| Li H 2021 | GA | LIA (ropivacaine 0.25%, 80 mL) | Celecoxib 200 mg BID (pre-op D−2 to 2 wk post-op) | None specified | IV morphine 5 mg PRN (VAS >6) |
| Rienstra W 2021 | NR (local protocols) | NR | Ongoing pre-existing analgesics continued; gabapentinoids prohibited perioperatively | NR | Unrestricted (except gabapentinoids) |
| Yuan M 2022 | GA | LIA (ropivacaine 0.25%, 80 mL) | Celecoxib 200 mg BID (pre-op D−2 to 3 wk post-op) | IV TXA (pre-incision + post-op ×2); no tourniquet | Oxycodone PO or morphine SC PRN (VAS >6) |
| YaDeau JT 2022 | Spinal-epidural | ACB + IPACK (bupivacaine); LIA (bupivacaine/epinephrine/methylprednisolone) | PCM 1 g IV × 4 then PO q8h; ketorolac IV → meloxicam 15 mg/d PO; SR oxycodone 10 mg pre-op | Tourniquet; IV ketamine 30–50 mg; IV dexamethasone 4 mg | PO oxycodone 5–10 mg PRN |
| Ding 2023 | GA | LIA (ropivacaine 0.25%, 80 mL) | Celecoxib 200 mg q12h; IV dexamethasone 10 mg ×2 | None specified | IV morphine 5 mg PRN (VAS >6) |
| Imani 2023 | Spinal (bupivacaine + fentanyl) | None | No scheduled background analgesics | None specified | IV PCM 1 g (VAS >3) |
| Inamullah 2023 | Regional (unspecified) | NR | NSAIDs pre-op (no standardized schedule); no post-op protocol described | NR | None specified |
| Pinsornsak 2024 | Spinal + continuous epidural (24 h) | LIA cocktailᵃ | Parecoxib IV 40 mg q12h (48 h) → celecoxib 200 mg/d PO; PCM 1 g q8h; pre-op: celecoxib, PCM, pregabalin 75 mg | Tourniquet; IV dexamethasone 8 mg | IV morphine 3 mg q4h PRN (VAS ≥4); tramadol 50 mg PO PRN after discharge |
| Rajani 2024 | Spinal (bupivacaine + buprenorphine) | Adductor canal infiltrationᵇ | IV PCM 1 g q8h (day of surgery); etoricoxib 60 mg q8h PO from POD1 to wk 4 | IV TXA 10 mg/kg; tourniquet (available, not used) | Etoricoxib 60 mg SOS (VAS ≥5) |

ACB, adductor canal block; BID, twice daily; CSE, combined spinal-epidural; GA, general anesthesia; IPACK, interspace between the popliteal artery and posterior knee capsule block; IV, intravenous; LIA, local infiltration analgesia; NR, not reported; PCA, patient-controlled analgesia; PCM, paracetamol; PCEA, patient-controlled epidural analgesia; POD, postoperative day; PO, oral; PRN, as needed; SC, subcutaneous; SOS, as needed; SR, sustained-release; TXA, tranexamic acid; VAS, visual analogue scale. ᵃ Pinsornsak 2024 LIA cocktail: bupivacaine 0.5% 100 mg, adrenaline 0.6 mg, ketorolac 30 mg, tranexamic acid 1 g, morphine 5 mg in 100 mL normal saline. ᵇ Rajani 2024 adductor canal infiltration: bupivacaine 0.5% 20 mL, fentanyl 200 µg, clonidine 50 µg, methylprednisolone acetate, ketorolac; administered through the incision site.
